# Unraveling the Drivers of Continuous Summer Ozone Pollution Episodes in Bozhou, China: Toward Targeted Control Strategies

**DOI:** 10.3390/toxics14010037

**Published:** 2025-12-29

**Authors:** Ke Wu, Xuezhong Wang, Dandan Zhang, Hong Li, Fang Bi, Zhenhai Wu, Fanxiu Li, Wanghui Chu, Cong An

**Affiliations:** 1 State Key Laboratory of Environmental Criteria and Risk Assessment, Chinese Research Academy of Environmental Sciences, Beijing 100012, China; 2 College of Chemistry and Environmental Engineering, Yangtze University, Jingzhou 434023, China; 3 Institute of Surface-Earth System Science, School of Earth System Science, Tianjin University, Tianjin 300072, China; 4 College of Mechanical Engineering, Beijing Institute of Petrochemical Technology, Beijing 102617, China; 5 Environmental Standard Institute, Ministry of Ecology and Environment, Chinese Research Academy of Environmental Sciences, Beijing 100012, China; 6 Chinese Academy for Environmental Planning, Beijing 100012, China

**Keywords:** ozone pollution process, causes and mechanisms, atmospheric oxidation capacity, sources apportionment, Bozhou, China

## Abstract

Given the deteriorating situation of ambient ozone (O_3_) pollution in some areas of China, understanding the mechanisms driving O_3_ formation is essential for formulating effective control measures. This study examines O_3_ formation mechanisms and RO*_x_* (OH, HO_2,_ and RO_2_) radical cycling driven by photochemical processes in Bozhou, located at the junction of Jiangsu–Anhui–Shandong–Henan (JASH), a region heavily affected by O_3_ pollution, by applying a zero-dimensional box model (Framework for 0-Dimensional Atmospheric Modeling, F0AM) coupled with the Master Chemical Mechanism (MCM v3.3.1) and Positive Matrix Factorization (PMF 5.0) to characterize O_3_ pollution, identify volatile organic compound (VOC) sources, and quantify radical budgets during pollution episodes. The results show that O_3_ episodes in Bozhou mainly occurred in June under conditions of high temperature and low wind speed. Oxygenated volatile organic compounds (OVOCs), alkanes, and halocarbons were the dominant VOCs groups. The CH_3_O_2_ + NO reaction accounted for 24.3% of O_3_ production, while photolysis contributed 68.7% of its removal. Elevated VOCs concentrations in Bozhou were largely maintained by anthropogenic sources such as vehicle exhaust, solvent utilization, and gasoline evaporation, which collectively enhanced O_3_ production. The findings indicate that O_3_ formation in the region is primarily regulated by NO*_x_* availability. Therefore, emission reductions targeting NO*_x_*, along with selective control of OVOCs and alkenes, would be the most effective strategies for lowering O_3_ levels. Model simulations further highlight Bozhou’s strong atmospheric oxidation capacity, with OVOC photolysis identified as the dominant contributor to RO*_x_* generation, accounting for 33% of the total. Diurnal patterns were evident: NO*_x_*-related reactions dominated radical sinks in the morning, while HO_2_ + RO_2_ reactions accounted for 28.5% in the afternoon. By clarifying the mechanisms of O_3_ formation in Bozhou, this study provides a scientific basis for designing ozone control strategies across the JASH junction region. In addition, ethanol was not directly measured in this study; given its potential to generate acetaldehyde and affect local O_3_ formation, its possible contribution introduces additional uncertainty that warrants further investigation.

## 1. Introduction

Since the implementation of the “Air Pollution Prevention and Control Action Plan” and the “Three-Year Action Plan to Win the Blue Sky Defense War” [1,2], significant progress has been achieved in mitigating PM_2.5_ pollution across China. However, the rising trend in O_3_ concentrations has not been effectively mitigated [3,4]. From 2015 to 2019, China experienced a pronounced increase in O_3_ pollution. The proportion of days dominated by O_3_ exceedance increased from 16.9% to 41.8%, and 103 cities exceeded the national O_3_ standard [5,6]. The observed pattern aligns with broader global trends, where background O_3_ levels have shown a steady upward trajectory over recent decades, indicative of a strengthened atmospheric oxidizing environment. Long-term monitoring supports this rise; for instance, Sicard [7] documented average annual O_3_ growth rates of about 0.15 ppbv in background regions and nearly double that, 0.31 ppbv, in urban environments. At the hemispheric scale, background O_3_ continues to increase, providing important context for regional pollution. In the mid-latitudes of the Northern Hemisphere, surface O_3_ exhibited an average annual growth rate of about 1% [8], while five of the seven long-term monitoring sites in the Southern Hemisphere reported decadal mean increases of 0.5–2 ppbv [9]. This large-scale enhancement of background O_3_ has also been reflected in China, where O_3_ concentrations have shown a persistent upward trend with pronounced spatial heterogeneity. At the Waliguan global background station in western China, O_3_ increased at an average rate of 0.2 ± 0.3 ppbv/year [10]. Long-term records further indicate significant increases across both diurnal and seasonal cycles, with growth rates of 0.24 ppbv/year during daytime, 0.28 ppbv/year at night, 0.24, 0.22, 0.29, and 0.13 ppbv/year in spring, summer, autumn, and winter, respectively [10,11,12,13]. Faster increases have been observed in regions with intensive anthropogenic activity and dense urbanization. For example, the Taishan site on the North China Plain recorded a summer O_3_ increase of 2.2 ± 1.2 ppbv/year [10], while the Hong Kong site reported a rate of 0.54 ± 0.49 ppbv/year [14]. Overall, these findings demonstrate a clear and sustained rise in O_3_ levels in China over recent decades, driven by both regional photochemistry and enhanced hemispheric background O_3_.

The junction of Jiangsu–Anhui–Shandong–Henan (JASH) provinces, situated between the Beijing–Tianjin–Hebei region and the Yangtze River Delta, also experiences severe O_3_ pollution. However, research on O_3_ formation mechanisms in China has primarily concentrated on major urban clusters, including the Pearl River Delta [15,16], the Yangtze River Delta [17], the North China Plain [18], and the Sichuan–Chongqing region [19,20,21]. In many urban settings, O_3_ production is primarily constrained by the availability of volatile organic compounds (VOCs), while suburban and rural regions tend to be governed by NO*_x_* limitations or exist within an intermediate sensitivity regime. Modeling results from Nanjing highlight this contrast, showing that on days when O_3_ levels surpassed the standard, the atmosphere exhibited a substantially higher oxidative capacity, reflected in OH and HO_2_ concentrations that were 1.6- and 1.7-fold greater than those on non-exceedance days [22]. Similarly, Wang et al. [23], using the Photochemical Box Model–Master Chemical Mechanism (PBM-MCM), reported that the South China Sea region operates under a transitional regime between VOC and NO*_x_* limitation on non-exceedance days, but shifts to VOC-limited conditions on O_3_ exceedance days. During exceedance days, enhanced photochemical cycling led to an average daytime net O_3_ production rate 3.2 times higher than on non-exceedance days. These results highlight that radical concentrations are influenced not only by the composition and abundance of VOCs but also by solar radiation intensity, both of which jointly regulate ambient O_3_ levels. Lyu et al. [18] further investigated RO*_x_* radical budgets and O_3_ formation, identifying VOC-limited conditions in Wuhan during autumn, where HONO played a major role in enhancing atmospheric oxidative capacity. Although recent studies have addressed the characteristics of O_3_ precursors [24], photochemical reaction mechanisms [25], and the influence of meteorology on O_3_ pollution [26], the mechanisms of O_3_ formation in the JASH junction remain insufficiently studied and poorly understood.

Bozhou, located at the JASH junction, possesses a strategically advantageous location and a distinctive industrial structure. It serves as a major national distribution center for traditional Chinese medicine and a renowned hub of the liquor industry. However, with the rapid progression of industrialization and urbanization, the city has been facing increasingly severe air pollution challenges. This study integrated meteorological observations, routine air pollutant measurements, and VOC monitoring at the Bozhou University site to investigate local O_3_ pollution. In parallel, the F0AM model coupled with the MCM was employed to simulate O_3_ formation and perform a comprehensive budget analysis. The findings aim to provide a scientific foundation for designing targeted strategies to mitigate O_3_ pollution in Bozhou and the surrounding region.

## 2. Materials and Methods

### 2.1. Data Sources

The Air quality and meteorological data were obtained from the China National Environmental Monitoring Center (http://www.cnemc.cn/sssj/ (accessed on 25 July 2025)) and the Anhui Province Air Quality Forecasting and Early Warning Platform. The dataset includes hourly measurements of six conventional air pollutants (PM_2.5_, PM_10_, NO_2_, SO_2_, CO, and O_3_), together with key meteorological parameters such as wind speed, wind direction, temperature, relative humidity, and atmospheric pressure. VOCs were measured at a comprehensive atmospheric supersite located at Bozhou University, which functions as a national intensive observation station and provides high-resolution in situ VOC observations. VOC measurements were conducted using an online gas chromatograph system (TH-300B, Wuhan Tianhong Instrument Co., Ltd., Wuhan, China) equipped with a GC–FID/MS configuration (7890B GC/5977B MSD, Agilent Technologies Inc., Santa Clara, CA, USA). Instrument operation and calibration followed national technical specifications (HJ/T 193-2005 and HJ 1010-2018; [27,28]), with full details provided in Supplementary Text S2. A map showing the study area and the location of the supersite is provided in the Supplement Figure S1. Pollution episodes were identified according to the China National Ambient Air Quality Standard. A pollution event was defined when either the 24 h average PM_2.5_ concentration exceeded 75 µg/m^3^ or the maximum daily 8 h average (MDA8) O_3_ exceeded 160 µg/m^3^ (≈75 ppbv), corresponding to the Grade II limits of GB 3095-2012 [29]. These observational datasets were subsequently used to initialize and constrain the zero-dimensional OBM, while detailed model configuration procedures are described in the Text S3.

### 2.2. Ozone Formation Potential

Ozone Formation Potential (OFP) is an indicator used to assess the capacity of VOCs to generate O_3_ through photochemical reactions in the troposphere, helping to identify the species that contribute most significantly to O_3_ formation. The OFP of individual VOC species was calculated using the following formula:

(1) $$OFP_{i}=VOCs_{i}\times MIR_{i}$$

where *VOC*s*_i_* is the ambient mass concentration of species *i* (μg/m^3^), and *MIR_i_* is its maximum incremental reactivity (gO_3_/gVOCs) [30]. The MIR coefficient represents the amount of ozone formed per unit mass of VOC under high-NO*_x_* conditions and is a widely applied metric for evaluating the relative ozone-forming potential of VOCs. The MIR values used in this study were adopted from Carter’s reactivity scale, which was established based on detailed photochemical box-model simulations and has been extensively employed in ozone formation assessments [31]. It should be noted that IR values derived from the OBM may differ from MIR- or MOIR/EBIR-based ozone formation potentials due to differences in chemical mechanisms and NO*_x_* regimes. A direct comparison with published reactivity scales would be valuable for assessing regime dependence and linking local photochemical responses to widely used metrics, and is suggested as a topic for future research.

### 2.3. Positive Matrix Factorization Model

The Positive Matrix Factorization (PMF) model, developed by the U.S. Environmental Protection Agency (EPA), has been extensively applied to identify sources of PM_2.5_ and VOCs. It is important to note that the source profiles resolved by PMF are derived from statistical covariance structures within the input dataset. As a result, individual factors may represent mixed sources or reflect co-emitted pollutants originating from distinct but temporally correlated activities. Factor identification and naming were carried out by combining chemical marker analysis with previous knowledge of regional emission characteristics.

The PMF model is described by the following equation:

(2) $$X_{ij}=\sum_{k=1}^{p}g_{ik}f_{kj}+e_{ij}$$

where *X_ij_* is the concentration of species *j* in sample *i*, in ppb; *g_ik_* is the contribution of source *k* to sample *i*; *f_ik_* is the source profile of species *j* in source *k*; *e_if_* is the residual matrix of species *j* in sample *i*; and *p* is the number of factors. The PMF model primarily minimizes the objective function *Q* [32], which is defined as:

(3) $$Q=\sum_{i=1}^{n}\sum_{j=1}^{m}{\left(\frac{e_{ij}}{u_{ij}}\right)}^{2}$$

where *n* is the number of samples, *m* is the number of species, and *u_ij_* represents the uncertainty of species in the samples.

When applying PMF to identify VOC sources, two input files are required: one containing the concentrations of the species (*Conc*.) and another containing their associated uncertainties (*Unc.*) [32,33]. The uncertainty of samples is calculated as follows:

(4) $$Unc.=\left\{\begin{array}{cc} \sqrt{{\left(Error\;Fraction\times Conc.\right)}^{2}+{\left(0.5\times \mathrm{MDL}\right)}^{2}} & (Conc.>\mathrm{MDL})\\ 5\times \mathrm{MDL}/6 & (Conc.\leq \mathrm{MDL}) \end{array}\right.$$

where MDL is the method detection limit. For concentrations below the MDL, a value equal to one-half of the MDL is substituted.

### 2.4. Observation-Based Model

To better understand the chemical mechanisms driving O_3_ pollution in Bozhou and to assess how O_3_ responds to reductions in its precursors, this study employed the observation-based model (OBM) F0AM to simulate atmospheric chemical processes. F0AM is a zero-dimensional box model constrained by observational data and driven by the MCM v3.3.1 [34,35,36]. The mechanism implemented in this study incorporates 5733 chemical species and 16,940 reactions, providing a comprehensive framework for simulating complex atmospheric chemistry. Details on model initialization, boundary layer assumptions, and the limitations of the F0AM simulations are presented in Supplementary Text S3. The input dataset for the F0AM model included the supersite’s geographic coordinates (latitude, longitude, and elevation), the observation period, hourly meteorological parameters (temperature, pressure, and relative humidity), concentrations of routine air pollutants (NO_2_, NO, SO_2_, CO, and O_3_), and VOC speciation data. Since photolysis rates were not directly measured, they were estimated using the solar zenith angle parameterization provided within the MCM framework [34,36]. To evaluate the impacts of precursor emissions on ozone formation, multiple reduction scenarios were simulated. The model was further used to quantify ozone production rates, hydroxyl radical (OH) reactivity, and the primary pathways of production, recycling, and termination of RO*_x_* radical species. OH reactivity was assessed based on its reaction kinetics with various atmospheric compounds, including VOCs, NO*_x_*, CO, SO_2_, nitric acid (HNO_3_), and peroxynitric acid (HO_2_NO_2_).

The primary sources of OH, HO_2_, and peroxy radicals (RO_2_) include the photolytic decomposition of ozone, nitrous acid (HONO), acetaldehyde, and other OVOCs, along with reactions between VOCs and ozone or nitrate radicals. To evaluate the chemical budget of reactive RO*_x_* radicals, following the approach of Wang et al. [37] and Xue et al. [38], the relevant reactions are categorized into three groups: major production, recycling, and removal pathways. Furthermore, the chemical budget of O_3_ is analyzed using a modeling approach. The ozone production rate, P(O_3_), is defined as the sum of the reaction rates of HO_2_ with NO and RO_2_ with NO (Equation (5)). The O_3_ loss rate, L(O_3_), is determined by adding the reaction rates of O_3_ photolysis and its reactions with OH, HO_2_, and NO_2_ (Equation (6)). The net O_3_ production rate is then obtained as the difference between P(O_3_) and L(O_3_) (Equation (7)). For clarity, only the key processes controlling O_3_ formation and loss are briefly summarized here. The kinetic expressions in Equations (5) and (6) are derived from the MCM, and the dominant pathways involve HO_2_/RO_2_–NO interactions and NO_2_ photolysis for O_3_ production, as well as NO titration, photolysis, and radical-driven chemistry for O_3_ loss. Detailed descriptions of these chemical calculations and the complete reaction framework are provided in the Text S4 and can also be found in the referenced literature [38,39]. It is important to note the limitations of the F0AM simulation. First, variations in planetary boundary layer dynamics are not explicitly represented, and thus vertical mixing effects, particularly during day–night transitions, may not be fully captured. Second, deposition is treated in a simplified manner, approximated as a uniform dilution process rather than being independently parameterized, which introduces uncertainty in simulating the diurnal evolution of pollutant concentrations and radical budgets.

(5) $$P\left(O_{3}\right)=k_{1}\left[{\mathrm{HO}}_{2}\right][\mathrm{NO}]+\sum \left(k_{2}\left[{\mathrm{RO}}_{2}\right][\mathrm{NO}]\right)$$

(6) $$\begin{array}{cc} L\left(O_{3}\right) & =k_{3}\left[O{(}^{1}D)\right]\left[H_{2}O\right]+k_{4}\left[O_{3}\right]\left[\mathrm{OH}\right]\\ & +k_{5}\left[O_{3}\right]\left[{\mathrm{HO}}_{2}\right]+k_{6}\left[{\mathrm{NO}}_{2}\right]\left[O_{3}\right] \end{array}$$

(7) $$NetP\left(O_{3}\right)=P\left(O_{3}\right)-L\left(O_{3}\right)$$

The Empirical Kinetic Modeling Approach (EKMA) and Relative Incremental Reactivity (RIR) are valuable tools for examining the relationship between O_3_ concentrations and their precursors, including VOCs and NO*_x_*. These approaches provide a scientific basis for formulating effective O_3_ pollution control strategies. Owing to the highly nonlinear interactions among O_3_, VOCs, and NO*_x_*, RIR is particularly effective in quantifying the sensitivity of O_3_ formation to its precursors in a given region.

(8) $$\mathrm{RIR}(X)=\frac{\left[P\left(O_{3}\right)(X)-P\left(O_{3}\right)(X-\Delta X)\right]/P\left(O_{3}\right)(X)}{\Delta c(X)/c(X)}$$

In the equation, *X* denotes a precursor, *c(X)* indicates its observed concentration in the atmosphere, and *Δc(X)/c(X)* expresses the relative change resulting from variations in the volume fraction of precursor *X*. The subsequent term corresponds to the net O_3_ production simulated by the F0AM model. In this study, RIR values were calculated by reducing the volume fraction of each precursor by 20%.

To evaluate the reliability of the model simulations, the Index of Agreement (IOA) was applied, following the formulation provided by Liu et al. [40].

(9) $$IOA=1-\frac{\sum_{i=1}^{n}{\left(O_{i}-S_{i}\right)}^{2}}{\sum_{i=1}^{n}{\left(\left|O_{i}-\bar{O}|+|S_{i}-\bar{O}\right|\right)}^{2}}$$

where $S_{i}$ is the simulated value, $O_{i}$ is the observed value, $\bar{O}$ is the average observed value, and n is the number of samples. The IOA follows the standard formulation originally proposed by Willmott [41] and ranges from 0 to 1, with higher values representing stronger agreement between simulated and observed data. Previous atmospheric modeling studies suggest that IOA values in the range of 0.68–0.89 generally indicate satisfactory model performance [23,42]. In this study, the IOA was calculated as 0.87, which falls within the upper range of previously reported values, demonstrating that the F0AM model reliably reproduces the observed O_3_ variations and is therefore suitable for subsequent radical analysis (Figure S2).

## 3. Results and Discussion

### 3.1. Overview of Ozone Pollution

From April to August 2023, a total of 32 days were classified as polluted in Bozhou, accounting for 20.9% of the monitoring period. On the majority of these days, O_3_ was the primary pollutant, responsible for 81.3% of the recorded pollution events. The highest occurrence of O_3_ pollution was recorded in June, with 16 exceedance days. A particularly severe and persistent episode unfolded from 8 to 16 June, during which the maximum daily 8 h average O_3_ concentration (MDA8h O_3_) peaked at 135.4 ppbv on 10 June. Owing to its intensity and duration, this nine-day episode was selected for detailed analysis of O_3_ formation mechanisms.

During the O_3_ pollution episode in Bozhou, MDA8h O_3_ concentrations ranged from 94.3 to 135.4 ppbv, consistently exceeding the national secondary standard of 75 ppbv. O_3_ formation was primarily driven by precursor availability and meteorological conditions, with daytime photochemical reactions serving as the dominant mechanism for O_3_ accumulation. Meteorological conditions during this period were characterized by a maximum temperature of 38.6 °C, low average wind speeds of 1.4 ± 0.5 m/s, and moderate average relative humidity of 47.7 ± 17.3% (Table S1).

**Figure 1:**
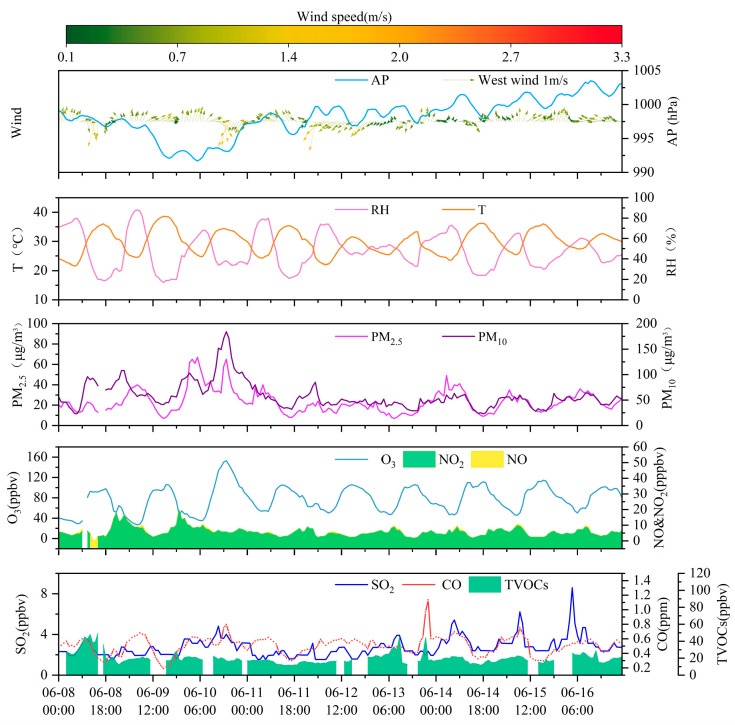
The time series analysis of trace gases, PM_2.5_, and meteorological parameters recorded at the Bozhou University site during the period from 8 to 16 June 2023.

Figure 1 presents a at the Bozhou University monitoring site, the concentrations of NO_2_, PM_2.5_, and O_3_ displayed similar temporal patterns and strong correlations, all statistically significant at the 0.01 level Figure S3a. The mean PM_2.5_ concentration was 23.5 ± 11.5 μg/m^3^, while the average NO_2_ concentration was 5.6 ± 2.8 ppbv. O_3_ levels were substantially high, with the maximum 1 h concentration reaching 152.7 ppbv.

**Figure 2:**
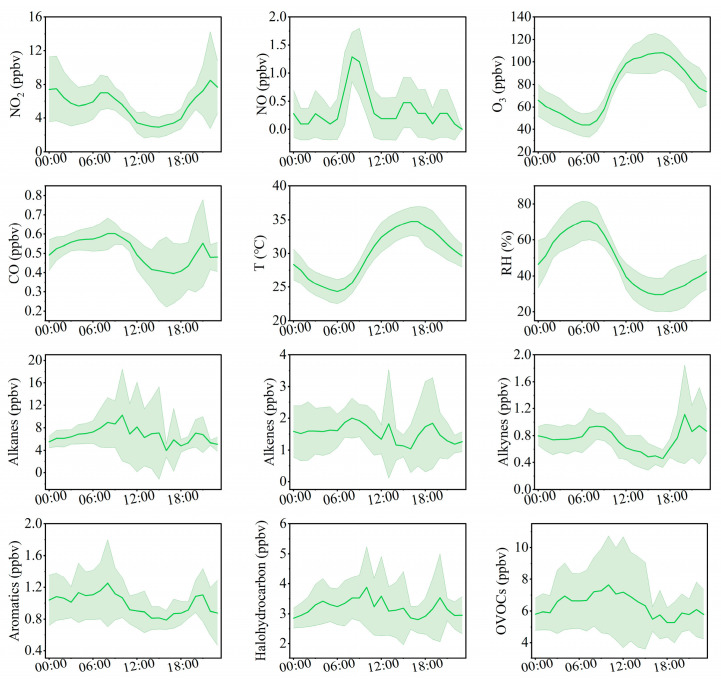
Average diurnal variation patterns of trace gases, meteorological parameters, and VOC components at the Bozhou University site from 8 to 16 June 2023 (green shading represents the standard deviation of the mean).

From a diurnal perspective (Figure 2), O_3_ concentrations remain at relatively low levels from nighttime until approximately 09:00 local time (LT), after which they increase and reach a maximum around 17:00 LT. In contrast, VOCs, NO*_x_*, and CO exhibit similar diurnal patterns, with higher concentrations during nighttime and lower concentrations during daytime. This behavior suggests that these precursors experience enhanced daytime removal, driven by a combination of photochemical processing and dilution associated with the daytime expansion of the planetary boundary layer, while accumulating at night under weaker mixing conditions. NO_2_ displays a distinct temporal pattern, with concentrations peaking around 08:00 LT, decreasing between 09:00 and 16:00 LT, and rising again during the evening and nighttime. This diurnal characteristic is closely related to local traffic management policies, under which heavy-duty diesel trucks are restricted from entering the urban area during daytime and primarily operate at night, thereby highlighting their critical contribution to nighttime NO*_x_* emissions. Bozhou’s location within the JASH junction, a major transport corridor connecting the Beijing–Tianjin–Hebei region with the Yangtze River Delta, further influences this trend. Road freight transport dominates the region, supported by a fleet of more than 2.4 million diesel trucks, which serve as a major source of NO*_x_* emissions. The combined influence of emission patterns and boundary layer dynamics [43,44] explains the observed diurnal variations. NO_2_ levels rose during morning and evening rush hours, followed by a pronounced decline at midday. NO exhibited sharp morning peaks that diminished rapidly. Across the study period, the mean daily concentrations were 5.4 ppbv for NO_2_, 0.4 ppbv for NO, and 0.5 ppmv for CO.

**Figure 3:**
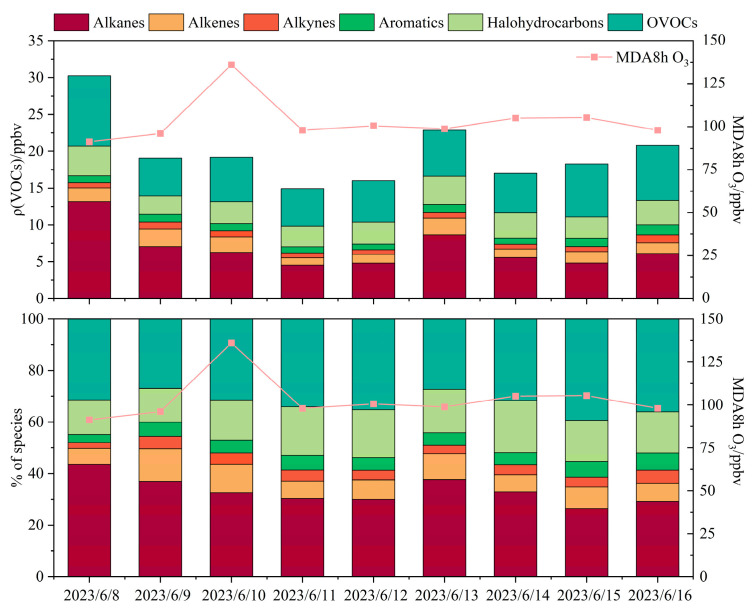
Variation in VOC components during the O_3_ pollution period.

Analysis of total VOCs (TVOCs) during the O_3_ pollution episode revealed that alkanes were the predominant group, with the highest daily average concentration (6.8 ppbv) and relative contribution (33.3%), followed by oxygenated volatile organic compounds (OVOCs), halogenated hydrocarbons, and alkenes (Figure 3). The daily average concentration and proportion of alkanes peaked on 8 June and subsequently showed a fluctuating decline. Alkynes and aromatic hydrocarbons exhibited a steady upward trajectory over the course of the episode, peaking on 16 June with daily mean concentrations of 1.1 ppbv (5.1%) and 1.4 ppbv (6.7%), respectively. Further diagnostic analysis showed an ethylene/ethane ratio of 0.5 ± 0.4 in Bozhou, which was significantly lower (*p* < 0.01) than that reported for aged air masses in Hong Kong (0.7 ± 0.1) (Figure S3b). This finding suggests that the elevated O_3_ levels in Bozhou were partly influenced by aged air masses, likely attributable to regional pollutant transport and/or enhanced in situ atmospheric oxidation [45].

Comparisons of TVOC concentrations during the pollution period indicate that Bozhou (19.8 ± 4.3 ppbv) has substantially lower levels than several major urban centers, including Beijing (44.2 ppbv), Chengdu (36.0 ppbv), Lanzhou (45.3 ppbv) [46], Nanjing (49.0 ppbv) [22], and Seoul (35.6 ppbv) [47]. The levels recorded in Bozhou exceeded those typically measured at background or remote sites, such as Taishan (8.8 ppbv) [48] and Waliguan (2.6 ppbv) [49]. They are, however, comparable to values reported in other medium-sized cities, including Luoyang (20.9 ppbv) [50] and Xi’an (19.0 ppbv) [51].

### 3.2. Ozone Formation Mechanisms

#### 3.2.1. Ozone Formation Sensitivity

**Figure 4:**
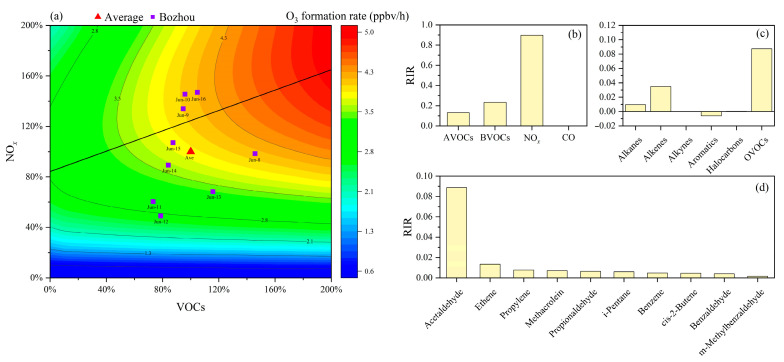
RIR values of O_3_ precursors and EKMA curve at Bozhou University site during the O_3_ pollution period. (**a**) EKMA curve during the O_3_ pollution period at Bozhou University site. (**b**) RIR values of 4 categories of O_3_ precursors; (**c**) RIR values of 5 categories of AVOCs components; (**d**) Top 10 VOCs species ranked by RIR values.

To investigate the relationship between O_3_ and its precursors, an EKMA curve analysis was conducted (Figure 4a). During the pollution episode, most volume fraction points were located below the ridge line, with a smaller proportion situated near the VOC–NO*_x_* transitional regime. This distribution indicates that local O_3_ formation was primarily constrained by NO*_x_* availability. The ridge line exhibited a slope of 0.4, indicating that the most effective and sustainable reduction in O_3_ would result from precursor control strategies aligned with this trajectory, implementing VOCs and NO*_x_* reductions at an approximate ratio of 0.4:1.

As shown in Figure 4b, NO*_x_* exhibits the highest RIR value (0.89), indicating that reductions in NO*_x_* emissions are most effective for mitigating local O_3_ pollution. This finding is consistent with the results presented in Section 3.3. CO contributes negligibly to O_3_ production, whereas VOCs play a more substantial role. Sensitivity analysis shows that anthropogenic VOCs (AVOCs) (excluding isoprene) have an RIR of 0.13, while biogenic VOCs (BVOCs), mainly isoprene, exert a stronger effect with an RIR of 0.23. The BVOC RIR in Bozhou is higher than values reported for Nanjing (0.12–0.13) [52] and is comparable to those observed in Shanghai [46].

As shown in Figure 4c, VOC components such as OVOCs, alkanes, and alkenes make substantial contributions to O_3_ formation, consistent with the findings presented in Section 3.4. The RIR-based ranking of the top 10 VOC species, illustrated in Figure 4d, further highlights their relative importance. Among these, acetaldehyde, ethylene, and propylene emerge as the most influential precursors, underscoring their priority for targeted control measures in Bozhou. A broader comparison with results from major regions in China (Table 1) indicates that O_3_ production in most urban areas is predominantly VOCs-limited, whereas suburban sites tend to be more sensitive to NO*_x_* emissions or exhibit mixed control regimes.

#### 3.2.2. Photochemical Ozone Formation Budget

**Figure 5:**
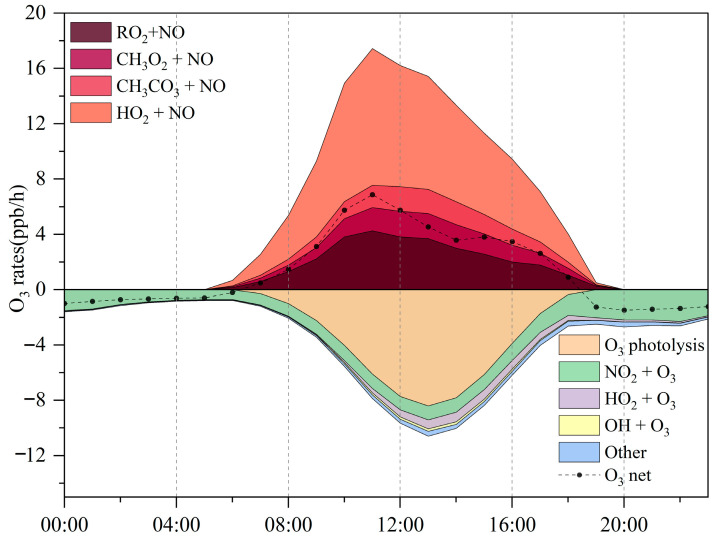
Diurnal variation of O_3_ production and consumption rates at the Bozhou University site during the O_3_ pollution period.

Simulation results (Figure 5) depict the production and loss processes of O_3_ during the pollution episode. The maximum net O_3_ production rate reached 9.6 ppbv/h, with an average daytime (06:00–18:00 LT) production rate of 9.8 ppbv/h and a daily mean of 4.2 ppbv/h. This daily average is slightly higher than that observed at the suburban Huaibei site (4.5 ppbv/h; [25]), but lower than values reported for Nanjing (4.9 ppbv/h; [22]) and Wuhan (6.2 ppbv/h; [65]), and comparable to Chengdu (4.2 ppbv/h; [66]). The O_3_ production rate peaks around 11:00 LT before declining sharply, a pattern likely linked to elevated morning NO levels that are subsequently consumed through photochemical reactions. O_3_ formation is driven primarily by the reactions of HO_2_ + NO and RO_2_ + NO, as shown in Figure 2.

The balance between O_3_ generation and loss during the pollution episode was illustrated using a stacked plot. Production rates climbed rapidly in the morning, reaching 17.4 ppbv/h near 11:00 LT, before tapering off. By 13:00 LT, production had dropped to 15.4 ppbv/h, coinciding with the highest consumption rate of 10.6 ppbv/h. The magnitude of this production rate is comparable to that reported for Paris, France (15.8 ppbv/h; [67]), but exceeds values observed in Berlin (8 ppbv/h; [68]), London (7.2 ppbv/h; [69]), and San Antonio (3.5 ppbv/h; [70]).

The observed variations highlight the strong influence of precursor emissions and prevailing photochemical conditions, characterized by elevated temperatures, low humidity, and stagnant air masses, on O_3_ formation, primarily through peroxy radical (HO_2_ and RO_2_) chemistry. Model simulations reveal that between 06:00 and 18:00 LT, the HO_2_ + NO pathway dominates, with an average reaction rate of 5.4 ± 2.9 ppbv/h, contributing 54.7% of total O_3_ production. The RO_2_ + NO pathway contributes slightly less, at 4.4 ± 1.3 ppbv/h (45.3%). More than 1000 RO_2_ radicals participate in these reactions, but the 12 most reactive species account for 77.9% of the total RO_2_ + NO reaction rate (3.5 ± 1.9 ppbv/h). The reaction between CH_3_O_2_ and NO accounted for the largest share of O_3_ production (24.3%), with CH_3_CO_3_ + NO contributing nearly as much (23.1%). On a daily mean basis, however, HO_2_ + NO dominated as the primary pathway driving O_3_ generation (Figure 5). However, O_3_ removal was controlled mainly by photolysis, responsible for 68.7% of losses, while the reaction of O_3_ with NO_2_ explained a further 19.1%.

### 3.3. Atmospheric Oxidation Capacity and Radical Chemistry

To assess the atmospheric oxidizing capacity during the O_3_ pollution episode, the F0AM model was applied to quantify the daytime (06:00–18:00 LT) RO*_x_* radical budget, including their sources and sinks. Figure S4 presents the simulated mean diurnal profiles of OH and HO_2_ concentrations. The maximum OH concentration reaches 7.53 × 10^6^ molec.cm^−3^, with an average value of 2.69 × 10^6^ molec.cm^−3^, while HO_2_ peaks at 1.08 × 10^9^ molec.cm^−3^, with an average of 4.04 × 10^8^ molec.cm^−3^.

The modeled daytime HO*_x_* (OH + HO_2_) concentrations in Bozhou are higher than those reported for Heshan in the Pearl River Delta [71] and are comparable to levels in Nanjing, Yangtze River Delta [22], where mean daytime OH and HO_2_ were 2.4 × 10^6^ molec.cm^−3^ and 4.7 × 10^8^ molec.cm^−3^, respectively. These results indicate that Bozhou exhibits a relatively strong atmospheric oxidizing capacity. Furthermore, the diurnal cycle of OH closely aligns with that of NO (Figure 2). The OH concentration peaks around midday (~11:00 LT), coinciding with the typical maximum driven by intense solar radiation, highlighting the critical role of photochemistry in enhancing OH production [72].

Photolysis of O_3_ represents the dominant source of RO*_x_* radicals, with an average production rate of 0.8 ± 0.7 ppbv/h during daylight hours. This pathway also plays a central role in OH radical formation. The subsequent generation of RO*_x_* is largely sustained through the production of HO_2_ and RO_2_ radicals. Among these, HO_2_ is primarily formed via the photolysis of OVOCs, whereas RO_2_ radicals originate mainly from VOC + O_3_ reactions and the photolysis of OVOCs.

**Figure 6:**
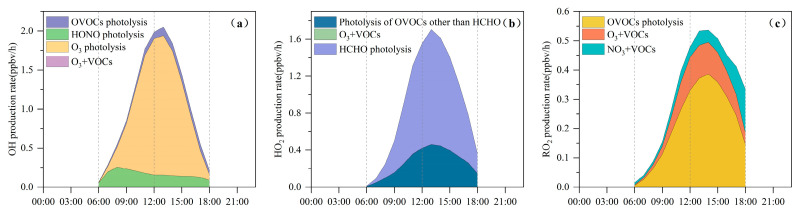
Average daytime primary production rates of (**a**) OH, (**b**) HO_2_, and (**c**) RO_2_ at the Bozhou University site during the O_3_ pollution period. (The O_3_ + VOCs contribution is too small to be visually apparent).

As illustrated in Figure 6a, O_3_ photolysis represents the principal source of primary OH radicals, with a peak rate of 1.8 ppbv/h at approximately 13:00 LT, accounting for 79% of the total primary OH production. HONO photolysis is the second most important contributor, reaching 0.3 ppbv/h (15%) around 08:00 LT. This early-morning peak is likely associated with traffic emissions and the nocturnal buildup of HONO [43].

**Figure 7:**
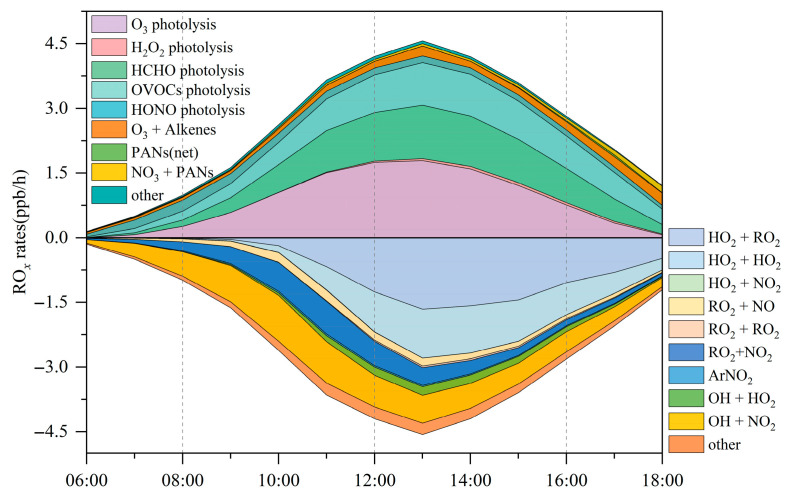
Daily fluctuations in RO*_x_* generation and consumption rates at the Bozhou University site during the O_3_ pollution period.

Regarding radical recycling, the HO_2_ + NO reaction is identified as the dominant secondary source of OH, contributing 5.4 ± 2.9 ppbv/h (Figure 7). The primary sinks of OH are reactions with VOCs (2.8 ± 1.6 ppbv/h), CO (2.6 ± 1.9 ppbv/h), and NO_2_ (2.4 ± 1.1 ppbv/h). The OH+O_3_ reaction constitutes only a minor loss pathway, with a rate of 0.1 ± 0.07 ppbv/h.

Figure 6b shows that HCHO photolysis is the dominant daytime source of HO_2_, with an average production rate of 0.63 ± 0.43 ppbv/h. The photolysis of other OVOCs contributes an additional 0.3 ± 0.12 ppbv/h. The OVOC photolysis rate in Bozhou is substantially lower than values reported for major urban environments such as Hong Kong [38] and Beijing [73]. Beyond photolytic processes, reactions of RO_2_ + NO (2.4 ± 1.3 ppbv/h) and OH + CO (1.8 ± 0.9 ppbv/h) also constitute important HO_2_ sources, as shown in Figure 7. The principal sink of HO_2_ is its reaction with NO (5.4 ± 2.9 ppbv/h), followed by self-reaction (HO_2_ + HO_2_), which occurs at 0.5 ± 0.4 ppbv/h.

In Figure 6c, the photolysis of OVOCs emerges as the dominant source of RO_2_ radicals, with an average production rate of 0.2 ± 0.1 ppbv/h, accounting for 66.6% of total primary RO_2_ formation. The reaction of unsaturated VOCs with O_3_ is the second major contributor, responsible for 19.7% of RO_2_ production. The recycling of OH through reactions with VOCs is 5.5 times greater than the combined yield of primary RO_2_ sources, underscoring the critical role of radical propagation in sustaining RO_2_ levels. The primary sink of RO_2_ is the HO_2_ + RO_2_ reaction, occurring at a rate of 0.7 ± 0.6 ppbv/h, and is largely governed by cross-reactions within the RO*_x_* radical family.

Therefore, the production of RO*_x_* radicals (P(RO*_x_*)) is dominated by photolytic processes. Among these, the photolysis of HCHO, OVOCs, HONO, and H_2_O_2_ contributes 25.6%, 22.8%, 6.5%, and 1.0% of P(RO*_x_*), respectively. The reaction of NO_3_ with VOCs serves only as a minor source, primarily for RO_2_ generation. The results highlight that OVOCs, comprising both directly emitted species and secondary carbonyls produced via VOC oxidation, serve as key initiators of atmospheric radical chemistry, exerting strong control over the primary radical budget. Consistently, a recent study in Huaibei City reported that OVOC photolysis accounted for 33% of RO*_x_* production [25].

RO*_x_* radicals are ultimately removed from the atmosphere through deposition processes involving species such as H_2_O_2_, RONO_2_, and ROOH. Throughout the monitoring period, the atmospheric profile exhibited a distinct daily cycle, with NO levels peaking during the morning hours before steadily diminishing as the day progressed. The reported reaction rates of peroxy radicals with NO range from 7.7 × 10^−12^ to 34 × 10^−12^ cm^3^s^−1^, while their self-reaction rates vary between 5.2 × 10^−12^ and 37 × 10^−12^ cm^3^s^−1^ [74]. As shown in Figure 7, RO*_x_* termination in the morning is dominated by reactions with NO*_x_*, whereas in the afternoon, self-combination of peroxy radicals becomes the principal sink. Daytime analysis indicates that HO_2_ + RO_2_, OH + NO_2_, HO_2_ + HO_2_, and RO_2_ + NO_2_ account for 28.5%, 22.7%, 19.8%, and 12.3% of the total RO*_x_* sinks, respectively. These results are consistent with previous findings showing that in low-NO*_x_* environments, self- and cross-reactions of peroxy radicals dominate termination, leading to substantial production of peroxides (H_2_O_2_ and ROOH). These peroxides can recycle radicals efficiently, generating OH with yields of up to 80% [75]. This behavior contrasts with high-NO*_x_* regions, where RO*_x_* termination is largely governed by reactions NO*_x_* with (particularly OH + NO_2_ and RO_2_ + NO) [76]. In the present study, these pathways contributed only 17.1% of the total RO*_x_* sinks, a result consistent with observations in suburban Beijing [73], the Yangtze River Delta [77], suburban Hong Kong [38], and urban Xiamen [78].

**Figure 8:**
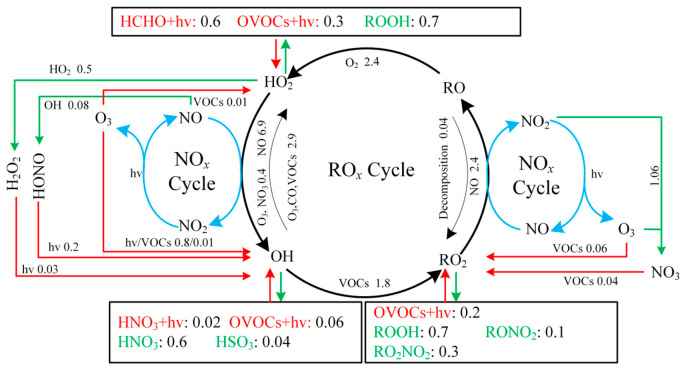
Diurnal average RO*_x_* budget from 06:00 to 18:00 LT during the O_3_ pollution period at the Bozhou University site. (The red, green, and black lines represent the production, consumption, and recycling pathways of radicals, respectively, in ppbv/h).

Figure 8 presents the average daytime RO*_x_* budget. Radical termination is dominated by cross-reactions among radicals, with HO_2_ + HO_2_ and HO_2_ + RO_2_ identified as the principal sinks. In Bozhou, the average daytime loss rates are 0.5 ± 0.4 ppbv/h for HO_2_ + HO_2_ and 0.7 ± 0.6 ppbv/h for HO_2_ + RO_2_, while RO*_x_* + NO*_x_* contributes 0.7 ± 0.4 ppbv/h. Compared with Bozhou, the RO*_x_* + NO*_x_* termination rates reported in Changzhou, Hong Kong, and Xiamen are more than twice as high, whereas Bozhou’s rate is more consistent with those observed in Huaibei and Dongying. These differences highlight the sensitivity of O_3_ production to NO*_x_* concentrations, underscoring the constraining role of NO*_x_* under VOC-rich conditions. Consistent with observations from other regions, the primary propagation pathway in Bozhou is the conversion of HO_2_ to OH. Among these processes, the HO_2_ + NO reaction (5.4 ± 2.9 ppbv/h) dominates OH production, exceeding the combined contributions from HONO and O_3_ photolysis by more than a factor of five. This finding emphasizes the critical role of NO*_x_* in sustaining atmospheric oxidation capacity. During VOC oxidation, OH radicals are rapidly consumed. Approximately 25.1% of OH reacts with VOCs, producing RO_2_ at a rate of 1.8 ± 1.0 ppbv/h, while 38.6% reacts with CO, O_3_, or HCHO to generate HO_2_ at 2.7 ± 1.5 ppbv/h. The reaction rate of OH with VOCs is nearly six times greater than the total primary production of RO_2_, highlighting the strong coupling between OH reactivity and secondary radical formation.

Furthermore, the reaction between RO_2_ and NO is the dominant pathway for HO_2_ production, contributing 2.4 ± 1.3 ppbv/h. Both RO_2_ + NO and HO_2_ + NO represent the most rapid propagation processes, facilitating the formation of NO-related RO and OH radicals, along with the byproduct O_3_. These propagation pathways clearly govern the overall production of OH, HO_2_, and RO_2_ radicals, consistent with the results of previous studies (Table S4).

### 3.4. Sources Apportionment of VOCs

To elucidate the major anthropogenic sources influencing O_3_ formation, a PMF model was applied to apportion VOC sources during the O_3_ episode. As illustrated in Figure S5, Factor 1 exhibited strong contributions from 2,2,4-trimethylpentane, methylcyclohexane, 2,3-dimethylpentane, and 3-methylhexane, compounds typically associated with industrial processes such as furniture manufacturing, feather product processing, printing, and home furnishings. These chemical profiles are consistent with industrial emissions [79,80]. Factor 2 was dominated by isopentane, a well-established tracer of gasoline evaporation [81,82], representing this source category. Factor 3 was characterized by high isoprene levels. In urban environments, isoprene is primarily of biogenic origin, and this factor was therefore identified as biogenic emissions [83]. Factor 4 included substantial contributions from long-chain alkanes and aromatics, such as n-heptane, 3-methylhexane, 2,3-dimethylpentane, 2,2,4-trimethylpentane, and methylcyclohexane, as well as toluene, ethylbenzene, and xylenes (m/p- and o-xylene). The printing industry is a known source of hexane, heptane, and toluene [84,85], while solvents such as toluene, ethylbenzene, and xylenes are widely used in coatings, paints, synthetic fragrances, adhesives, and cleaning agents [86,87]. Given Bozhou’s substantial number of alcoholic beverage and traditional Chinese medicine packaging printing facilities, this factor was attributed to solvent use emissions. Factor 5 was linked to vehicle exhaust emissions, as indicated by high levels of C_2_–C_5_ alkanes (e.g., propane, n-pentane, and n-butane), which are characteristic markers of traffic sources [88]. Further, NO*_x_* (introduced as a tracer gas in the model) showed its highest proportion (50%) in this factor, supporting its identification as a vehicular source [89].

**Figure 9:**
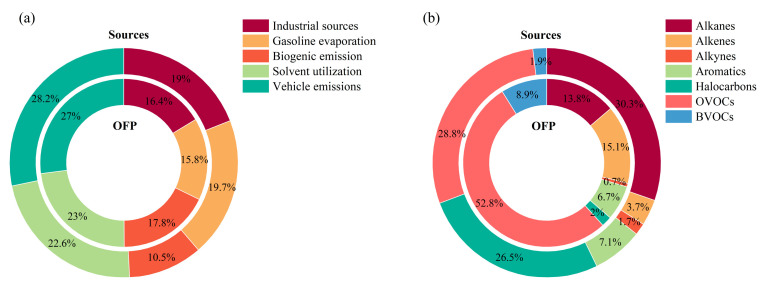
Percentages of different VOC sources and species groups to OFP at the Bozhou University site. (**a**) VOC emission sources; (**b**) VOC species groups.

Figure 9a illustrates the relative contributions of the five identified VOC sources at Bozhou University, expressed in terms of both concentration and OFP. Based on concentration, vehicle emissions constituted the largest share (28.2%), followed by solvent use (22.6%), gasoline evaporation (19.7%), industrial sources (19.0%), and biogenic emissions (10.5%). When assessed by OFP, vehicle emissions again dominated (27.0%), with solvent use (23.0%), biogenic emissions (17.8%), industrial sources (16.4%), and gasoline evaporation (15.8%) contributing in decreasing order. Taken together, these results indicate that vehicle emissions and solvent use are the primary contributors to O_3_ formation in Bozhou.

Figure 9b presents the composition of TVOCs and their respective contributions to OFP at the Bozhou University site. OVOCs represent the largest fraction of TVOCs and are also the dominant contributors to OFP, with acetaldehyde, propionaldehyde, and crotonaldehyde identified as the key species. Alkenes are the second-largest contributors, primarily driven by ethylene, propylene, and 1-butene, while alkanes rank third, with significant contributions from isopentane, n-butane, and propane. These results are consistent with findings in Xi’an [62], where OVOCs accounted for nearly 60% of total OFP.

During O_3_ pollution episodes, elevated temperatures and strong ultraviolet radiation can enhance production and emission of BVOCs such as isoprene and oxygenated VOCs from vegetation, thereby strengthening their role in O_3_ formation. In addition, elevated temperatures and drought conditions may further promote BVOC emissions (particularly monoterpenes and isoprene), potentially altering O_3_ sink pathways; however, this effect cannot be fully assessed in this study because monoterpenes were not measured due to instrumental constraints [90]. Although alkanes are present at relatively high concentrations, their contributions to OFP remain limited due to their comparatively low maximum incremental reactivity (MIR). Aromatic hydrocarbons, despite lower concentrations in Bozhou, generally exhibit higher reactivity and can dominate OFP in regions with extensive solvent use. For instance, in the Pearl River Delta, the proportion of aromatics in OFP has been reported to range from 47 to 65% in Shenzhen [91,92,93] and up to 70% in Guangzhou [94].

## 4. Conclusions

In this study, the characteristics of O_3_ pollution and the VOC composition in Bozhou were comprehensively analyzed. Using the F0AM model coupled with the MCM, the dominant processes driving O_3_ formation and removal were identified. O_3_ pollution events were found to occur predominantly in June, coinciding with high temperatures and low wind speeds. Among VOCs, OVOCs, alkanes, and halocarbons exhibited the highest concentrations and contributions. The CH_3_O_2_ + NO reaction was the most significant contributor to local O_3_ formation (24.3%), whereas O_3_ photolysis represented the dominant removal pathway (68.7%). Sensitivity analysis combined with the EKMA curve further revealed that O_3_ formation in Bozhou is NO*_x_*-controlled, and that reducing emissions of OVOCs and alkenes would be particularly effective in mitigating local O_3_ levels. Photolysis of OVOCs was also identified as a key driver of radical chemistry, accounting for 33% of RO*_x_* production. The removal of RO*_x_* radicals showed distinct diurnal patterns: in the morning, reactions with NO*_x_* dominated, while in the afternoon, self- and cross-reactions of peroxy radicals, the HO_2_ + RO_2_ pathway in particular, accounted for 28.5% of radical sinks. Source apportionment further indicated that vehicle emissions, solvent use, and gasoline evaporation were the major anthropogenic contributors during O_3_ pollution episodes. However, the role of ethanol emissions, particularly from local beverage industries, could not be assessed in this study due to limitations in ethanol measurement methods. While ethanol is a significant precursor for acetaldehyde, a compound with a major role in O_3_ formation, its potential contribution to O_3_ production should not be overlooked. Future studies and air quality management strategies should consider the regulation of ethanol emissions, especially from unregulated sources, to fully address O_3_ pollution concerns. These findings highlight the critical importance of targeting OVOCs, alkenes, and alkanes in emission control strategies to effectively manage O_3_ pollution in Bozhou. Therefore, future air quality management in Bozhou should further reinforce local control measures, with a particular focus on regulating motor vehicle emissions, solvent usage, and gasoline volatilization, while simultaneously restricting urban NO*_x_* emissions. This study reveals that O_3_ pollution in Bozhou and other cities at the junction of the JASH provinces is characterized by a NO*_x_*-limited regime, OVOC-dominated reactivity, and dual contributions from traffic and solvent-related sources. These results underscore the necessity of prioritizing NO*_x_* abatement, implementing targeted control of highly reactive VOCs, and enhancing cross-regional collaboration to achieve more effective mitigation of O_3_ pollution.

## Figures and Tables

**Table 1:** Summary of O_3_ formation sensitivity, key active VOCs species, and O_3_ formation mechanisms in major regions of China.

Region	Site	Type	O_3_ Formation Regime	Dominant VOCs	References
Beijing-Tianjin-Hebei Province and surrounding areas	Beijin	Urban	VOC-limited	Alkenes	[53]
	Jinan	Urban	VOC-limited	Alkenes	
	Baoding	Urban	VOC-limited	Aromatics	[54]
	Qingdao	Rural	Transition	Alkenes	[55]
	Yucheng	Rural	NO*_x_*-limited	BVOCs	
	Wangdu	Suburban	Transition	Alkenes	[56]
Yangtze River Delta region	Nanjing	Industrial	VOC-limited	Alkenes, Aromatics	[57]
	Shanghai	Urban	VOC-limited	Aromatics	[53]
		Rural	VOC-limited	Aromatics	
	Pudong	Urban	VOC-limited	Alkenes	[55]
	Xuzhou	Urban	VOC-limited	——	
	Yancheng	Urban	VOC-limited	——	
	Nantong	Urban	Transition	——	
	Dianshan Lake	Suburban	Transition	——	
Pearl River Delta region	Tung chung	Suburban	VOC-limited	——	[58]
	Shenzhen	Urban	VOC-limited	Aromatics	[59]
	Heshan	Rural	VOC-limited	Aromatics	
	Guangzhou	Urban	VOC-limited	——	[60]
	Huadu	Suburban	NO*_x_*-limited	——	
	Xinken	Suburban	NO*_x_*-limited	——	
Sichuan and Chongqing area	Chengdu	Urban	VOC-limited	Aromatics, Alkenes	[53]
	Shuangliu	Urban	VOC-limited	Aromatics	
	Pengzhou	Industrial	VOC-limited	Alkenes	
	Jinyun Mountain	Suburban	NO*_x_*-limited	Alkenes	[61]
Fenhe–Weihe River Plain	Xi’an	Urban	VOC-limited	——	[62]
	Qinling Mountains	Suburban	NO*_x_*-limited	——	
	Weinan	Urban	Transition	Alkenes	[55]
The junction of Jiangsu-Anhui-Shandong-Henan	Huaibei	Suburban	NO*_x_*-limited	OVOCs, Alkanes	[25]
	Dongying	Industrial	NO*_x_*-limited	Alkenes, OVOCs	[63]
		Rural	NO*_x_*-limited	Alkenes	[64]
	Bozhou	Urban	NO*_x_*-limited	OVOCs, Alkenes	This work

## Data Availability

The original contributions presented in this study are included in the article/Supplementary Material. Further inquiries can be directed to the corresponding author.

## Supplementary Materials

The following supporting information can be downloaded at: https://www.mdpi.com/article/10.3390/toxics14010037/s1, Text S1: Error estimation analysis of PMF results; Text S2: Online monitoring of VOCs: sampling, analysis, and quality control; Text S3: Model initialization and boundary layer assumptions in F0AM; Text S4: Mechanisms of O_3_ Formation and Depletion in Ambient Air; Text S5: Applicability and limitations of the OFP method and complementarity with RIR analysis; Table S1: Presents the hourly measurements of trace gas concentrations, PM_2.5_ levels, meteorological parameters, and total volatile organic compounds (TVOCs) recorded at the Bozhou University site during the period from 8 June to 16 June 2023; Table S2: VOCs species and the corresponding method detection limits (MDL, unit: ppbv) and MIR values; Table S3: VOC concentration measurements in Bozhou from 8 June to 16 June 2023 (units: pptv); Table S4: Summary of model reaction fluxes: Average reaction rates (ppbv/h) of radical formation, cross-reactions, and termination throughout the entire reaction process; Figure S1: Map of monitoring sites in Bozhou City; Figure S2: Comparison of simulated and observed O_3_ at the Bozhou University site from 8 June to 16 June 2023; Figure S3: (a) Correlation of NO_2_, PM_2.5_, and O_3_ concentrations at the Bozhou University site, (b) Correlation between ethylene and ethane; Figure S4: Average diurnal variation of OH and HO_2_ during simulated O_3_ pollution period at the Bozhou University site (shading represents the standard deviation of the mean across 6 simulations); Figure S5: Contributions of various species to the Bozhou University site during the O_3_ pollution period as analyzed by the PMF model; Figure S6: Top 10 species of OFP and their proportions at the Bozhou University site during the O_3_ pollution period. References [95,96,97,98,99,100,101] are cited in supplementary materials.

## References

[1] Zhang Q., Zheng Y., Tong D., Shao M., Wang S., Zhang Y., Xu X., Wang J., He H., Liu W. (2019). Drivers of improved PM_2.5_ air quality in China from 2013 to 2017. Proc. Natl. Acad. Sci. USA.

[2] Jiang X., Li G., Fu W. (2021). Government environmental governance, structural adjustment and air quality: A quasi-natural experiment based on the Three-year Action Plan to Win the Blue Sky Defense War. J. Environ. Manag..

[3] Chen Z., Chen D., Kwan M.-P., Chen B., Gao B., Zhuang Y., Li R., Xu B. (2019). The control of anthropogenic emissions contributed to 80% of the decrease in PM_2.5_ concentrations in Beijing from 2013 to 2017. Atmos. Chem. Phys..

[4] Feng Y., Ning M., Lei Y., Sun Y., Liu W., Wang J. (2019). Defending blue sky in China: Effectiveness of the “Air Pollution Prevention and Control Action Plan” on air quality improvements from 2013 to 2017. J. Environ. Manag..

[5] MEE (2020). China Ecological and Environmental Bulletin (2019).

[6] Wu Z., Wang Z., Zhang Q., Lu K., Li X., Hu J., Guo S., Xing J., Tong D., Wang D. (2022). Strategical research on refined regulations for regional air quality with climate synergy. Chin. J. Eng. Sci..

[7] Sicard P. (2021). Ground-level ozone over time: An observation-based global overview. Curr. Opin. Environ. Sci. Health.

[8] Vingarzan R. (2004). A review of surface ozone background levels and trends. Atmos. Environ..

[9] Cooper O.R., Schultz M.G., Schröder S., Chang K.-L., Gaudel A., Benítez G.C., Cuevas E., Fröhlich M., Galbally I.E., Molloy S. (2020). Multi-decadal surface ozone trends at globally distributed remote locations. Elem. Sci. Anthr..

[10] Xu W., Xu X., Lin M., Lin W., Tarasick D., Tang J., Ma J., Zheng X. (2018). Long-term trends of surface ozone and its influencing factors at the Mt Waliguan GAW station, China—Part 2: The roles of anthropogenic emissions and climate variability. Atmos. Chem. Phys..

[11] Zhu B., Akimoto H., Wang Z., Sudo K., Tang J., Uno I. (2004). Why does surface ozone peak in summertime at Waliguan?. Geophys. Res. Lett..

[12] Xu W., Lin W., Xu X., Tang J., Huang J., Wu H., Zhang X. (2016). 2016. Long-term trends of surface ozone and its influencing factors at the Mt Waliguan GAW station, China—Part 1: Overall trends and characteristics. Atmos. Chem. Phys..

[13] Liu N., Ma J., An X., Lin W., Xu W., Xu X., Li D., Li R. (2022). Source contributions and regional representativeness of surface ozone at atmospheric background stations in China. Trans. Atmos. Sci..

[14] Xue L., Wang T., Louie P.K.K., Luk C.W.Y., Blake D.R., Xu Z. (2014). Increasing external effects negate local efforts to control ozone air pollution: A case study of Hong Kong and implications for other Chinese cities. Environ. Sci. Technol..

[15] Hofzumahaus A., Rohrer F., Lu K., Bohn B., Brauers T., Chang C.C., Fuchs H., Holland F., Kita K., Kondo Y. (2009). Amplified trace gas removal in the troposphere. Science.

[16] Liu J., Wu D., Fan S., Liao Z.H., Deng T. (2017). Impacts of precursors and meteorological factors on ozone pollution in Pearl River Delta. China Environ. Sci..

[17] An J.L., Zhu B., Wang H.L., Li Y.Y., Lin X., Yang H. (2014). Characteristics and source apportionment of VOCs measured in an industrial area of Nanjing, Yangtze River Delta, China. Atmos. Environ..

[18] Lyu X.P., Wang N., Guo H., Xue L.K., Jiang F., Zeren Y.Z., Cheng H.R., Cai Z., Han L.H., Zhou Y. (2019). Causes of a continuous summertime O3 pollution event in Jinan, a central city in the North China Plain. Atmos. Chem. Phys..

[19] Chen D.Y., Zhou L., Wang C., Liu H.F., Qiu Y., Shi G.M., Song D.L., Tan Q.W., Yang F.M. (2022). Characteristics of ambient volatile organic compounds during spring O_3_ pollution episode in Chengdu, China. J. Environ. Sci-China.

[20] Lei Y., Wu K., Zhang X.L., Kang P., Du Y.S., Yang F.M., Fan J., Hou J.W. (2023). Role of meteorology-driven regional transport on O3 pollution over the Chengdu Plain, southwestern China. Atmos. Res..

[21] Li L., Li Z.L., Fang W.K., Wang X.C., Pu X., Wang L.T., Yuan R., Zhang W.D., Zhai C.Z. (2022). Speciated Emission Inventory of VOCs from Industrial Sources and Their Ozone Formation Potential in Chongqing. Environ. Sci..

[22] Luo L.T., Zhang Y.L., Lin Y.Q., Mozaffar A., Cao M.Y. (2024). Analysis of Photochemical Characteristics and Sensitivity of Atmospheric Ozone in Nanjing in Summer. Environ. Sci..

[23] Wang Y., Guo H., Zou S.C., Lyu X.P., Ling Z.H., Cheng H.R., Zeren Y.Z. (2018). Surface O_3_ photochemistry over the South China Sea: Application of a near-explicit chemical mechanism box model. Environ. Pollut..

[24] Ju T., Wu K., Zhang L., Wang G., Yan Y., Liu H., Li J., Chen X. (2022). A characteristic analysis of various air pollutants and their correlation with O_3_; in the Jiangsu, Shandong, Henan, and Anhui provinces of China. Sustainability.

[25] Wei N.N., Zhao W.X., Yao Y.C., Wang H.R., Liu Z., Xu X.Z., Rahman M., Zhang C.H., Fittschen C., Zhang W.J. (2023). Peroxy radical chemistry during ozone photochemical pollution season at a suburban site in the boundary of Jiangsu–Anhui–Shandong–Henan region, China. Sci. Total Environ..

[26] Wu K., Wang X.Z., Zhang D.D., Zhu H.L., Yan Y.X., Li F.X., Wu Z.H., Zheng Z.W., Gao Q.K. (2024). Evolution characteristics and typical pollution episodes of PM_2.5_ and O_3_ complex pollution in Bozhou City from 2017 to 2022. Environ. Sci..

[27] (2005). Automated Methods for Ambient Air Quality Monitorin.

[28] (2018). Specifications and Test Procedures for Ambient Air Quality Continuous Monitoring System with Gas Chromatography for Volatile Organic Compounds.

[29] (2012). Ambient Air Quality Standards.

[30] Atkinson R., Arey J. (2003). Atmospheric degradation of volatile organic compounds. Chem. Rev..

[31] Carter W.P.L. (1994). Development of Ozone Reactivity Scales for Volatile Organic Compounds. Air Waste.

[32] Brown S.G., Frankel A., Hafner H.R. (2007). Source apportionment of VOCs in the Los Angeles area using positive matrix factorization. Atmos. Environ..

[33] USEPA (2014). Positive Matrix Factorization (PMF) 5.0 Fundamentals and User Guide.

[34] Wolfe G.M., Marvin M.R., Roberts S.J., Travis K.R., Liao J. (2016). The framework for 0-D atmospheric modeling (F0AM) v3.1. Geosci. Model. Dev..

[35] Saunders S.M., Jenkin M.E., Derwent R.G., Pilling M.J. (2003). Protocol for the development of the Master Chemical Mechanism, MCM v3 (Part A): Tropospheric degradation of non-aromatic volatile organic compounds. Atmos. Chem. Phys..

[36] Jenkin M.E., Young J.C., Rickard A.R. (2015). The MCM v3.3.1 degradation scheme for isoprene. Atmos. Chem. Phys..

[37] Wang W.T., Gu C., Li L.M., Li X.Q., Zheng Z.S., Geng C.M., Wang X.L., Yang W. (2024). Photochemical mechanism and control strategy optimization for summertime ozone pollution in Yining City. Environ. Sci..

[38] Xue L.K., Gu R.R., Wang T., Wang X.F., Saunders S., Blake D., Louie P.K.K., Luk C.W.Y., Simpson I., Xu Z. (2016). Oxidative capacity and radical chemistry in the polluted atmosphere of Hong Kong and Pearl River Delta region: Analysis of a severe photochemical smog episode. Atmos. Chem. Phys..

[39] Xue L.K., Wang T., Gao J., Ding A.J., Zhou X.H., Blake D.R., Wang X.F., Saunders S.M., Fan S.J., Zuo H.C. (2014). Ground-level ozone in four Chinese cities: Precursors, regional transport and heterogeneous processes. Atmos. Chem. Phys..

[40] Liu X.F., Lyu X.P., Wang Y., Jiang F., Guo H. (2019). Intercomparison of O_3_ formation and radical chemistry in the past decade at a suburban site in Hong Kong. Atmos. Chem. Phys..

[41] Willmott C.J. (1981). On the Validation of Models. Phys. Geogr..

[42] Huang J.P., Fung J.C.H., Lau A.K.H., Qin Y. (2005). Numerical simulation and process analysis of typhoon-related ozone episodes in Hong Kong. J. Geophys. Res. Atmos..

[43] Hu B.Y., Liu T.T., Hong Y.W., Xu L.L., Li M.G., Wu X., Wang H., Chen J.H., Chen J.S. (2020). Characteristics of peroxyacetyl nitrate (PAN) in a coastal city of southeastern China: Photochemical mechanism and pollution process. Sci. Total Environ..

[44] Elshorbany Y.F., Kurtenbach R., Wiesen P., Lissi E., Rubio M., Villena G., Gramsch E., Rickard A.R., Pilling M.J., Kleffmann J. (2009). Oxidation capacity of the city air of Santiago, Chile. Atmos. Chem. Phys..

[45] Wang H., Lyu X., Guo H., Wang Y., Zou S., Ling Z., Wang X., Jiang F., Zeren Y., Pan W. (2018). Ozone pollution around a coastal region of South China Sea: Interaction between marine and continental air. Atmos. Chem. Phys..

[46] Liu X.F., Guo H., Zeng L.W., Lyu X.P., Wang Y., Zeren Y.Z., Yang J., Zhang L.Y., Zhao S.Z., Li J. (2021). Photochemical ozone pollution in five Chinese megacities in summer 2018. Sci. Total Environ..

[47] Song S.K., Shon Z.H., Kang Y.H., Kim K.H., Han S.B., Kang M.S., Bang J.H., Oh I. (2019). Source apportionment of VOCs and their impact on air quality and health in the megacity of Seoul. Environ. Pollut..

[48] Na K., Kim Y.P. (2001). Seasonal characteristics of ambient volatile organic compounds in Seoul, Korea. Atmos. Environ..

[49] Xue L.K., Wang T., Guo H., Blake D.R., Tang J., Zhang X.C., Saunders S.M., Wang W.X. (2013). Sources and photochemistry of volatile organic compounds in the remote atmosphere of western China: Results from the Mt. Waliguan Observatory. Atmos. Chem. Phys..

[50] Sun J., Shen Z.X., Wang R.N., Li G.H., Zhang Y., Zhang B., He K., Tang Z.Y., Xu H.M., Qu L.L. (2021). A comprehensive study on ozone pollution in a megacity in North China Plain during summertime: Observations, source attributions and ozone sensitivity. Environ. Int..

[51] Sun J., Shen Z.X., Zhang Y., Zhang Z., Zhang Q., Zhang T., Niu X.Y., Huang Y., Cui L., Xu H.M. (2019). Urban VOC profiles, possible sources, and its role in ozone formation for a summer campaign over Xi’an, China. Environ. Sci. Pollut. Res..

[52] Lu X.B., Wang M., Ding F., Yu Y.Y., Zhang Z.H., Hu K. (2023). Changes in O_3_-VOCs-NO*_x_* Sensitivity and VOCs Sources at an Urban Site of Nanjing Between 2020 and 2021. Environ. Sci..

[53] Jiang M.Q., Lu K.D., Su R., Tan Z.F., Wang H.L., Li L., Fu Q.Y., Zhai C.Z., Tan Q.W., Yue D.L. (2018). Ozone formation and key VOCs in typical Chinese city clusters. Chin. Sci. Bull..

[54] Wang M., Hu K., Chen W., Shen X., Li W., Lu X. (2020). Ambient non-methane hydrocarbons (NMHCs) measurements in Baoding, China: Sources and roles in ozone formation. Atmosphere.

[55] Lu H.X., Lyu X.P., Cheng H.R., Ling Z.H., Guo H. (2019). Overview on the spatial–temporal characteristics of the ozone formation regime in China. Environ. Sci. Process. Impacts.

[56] Han X., Zhu L.Y., Wang S.L., Meng X.Y., Zhang M.G., Hu J. (2018). Modeling study of impacts on surface ozone of regional transport and emissions reductions over North China Plain in summer 2015. Atmos. Chem. Phys..

[57] Zhang Y.X., An J.L., Wang J.X., Shi Y.Z., Liu J.D., Liang J.S. (2018). Source analysis of volatile organic compounds in the Nanjing industrial area and evaluation of their contribution to ozone. Environ. Sci..

[58] Cheng H.R., Guo H., Wang X.M., Saunders S.M., Lam S.H.M., Jiang F., Wang T.J., Ding A.J., Lee S.C., Ho K.F. (2010). On the relationship between ozone and its precursors in the Pearl River Delta: Application of an observation-based model (OBM). Environ. Sci. Pollut. Res..

[59] Wang T., Xue L.K., Brimblecombe P., Lam Y.F., Li L., Zhang L. (2017). Ozone pollution in China: A review of concentrations, meteorological influences, chemical precursors, and effects. Sci. Total Environ..

[60] Gao D.F., Zhang Y.H., Cao Y.Q. (2007). Process analysis of ozone formation in Guangzhou: Application of observation-based model. Res. Environ. Sci..

[61] Tan Z.F., Lu K.D., Jiang M.Q., Su R., Dong H.B., Zeng L.M., Xie S.D., Tan Q.W., Zhang Y.H. (2018). Exploring ozone pollution in Chengdu, southwestern China: A case study from radical chemistry to O_3_-VOC-NO*_x_* sensitivity. Sci. Total Environ..

[62] Guan X.Y., Lu K.D., Zhang N.N., Li X., Ma X.F., Yang X.P., Dong H.B., Chen S.Y., Cao J., Zeng L.M. (2021). Analysis of the photochemical characteristics and sensitivity of ozone pollution in Xi’an. Chin. Sci. Bull..

[63] Chen T.S., Xue L.K., Zheng P.G., Zhang Y.N., Liu Y.H., Sun J.J., Han G.X., Li H.Y., Zhang X., Li Y.F. (2020). Volatile organic compounds and ozone air pollution in an oil production region in northern China. Atmos. Chem. Phys..

[64] Sun X.Y., Zhao M., Shen H.Q., Liu Y., Du M.Y., Zhang W.J., Xu H.Y., Fan G.L., Gong H.L., Li Q.S. (2022). Ozone formation and key VOCs of a continuous summertime O_3_ pollution event in Ji’nan. Environ. Sci..

[65] Lu X.C., Chen N., Wang Y.H., Cao W.X., Zhu B., Yao T., Fung J.C.H., Lau A.K.H. (2017). Radical budget and ozone chemistry during autumn in the atmosphere of an urban site in central China. J. Geophys. Res. Atmos..

[66] Zhao Y.Y., Chen L.H., Li K.W., Han L.X., Zhang X., Wu X.C., Gao X., Azzi M., Cen K.F. (2020). Atmospheric ozone chemistry and control strategies in Hangzhou, China: Application of a 0-D box model. Atmos. Res..

[67] Michoud V., Kukui A., Camredon M., Colomb A., Borbon A., Miet K., Aumont B., Beekmann M., Durand-Jolibois R., Perrier S. (2012). Radical budget analysis in a suburban European site during the MEGAPOLI summer field campaign. Atmos. Chem. Phys..

[68] Mihelcic D., Holland F., Hofzumahaus A., Hoppe L., Konrad S., Müsgen P., Pätz H.-W., Schäfer H.J., Schmitz T., Volz-Thomas A. (2003). Peroxy radicals during BERLIOZ at Pabstthum: Measurements, radical budgets and ozone production. J. Geophys. Res. Atmos..

[69] Emmerson K.M., Carslaw N., Carslaw D.C., Lee J.D., McFiggans G., Bloss W.J., Gravestock T., Heard D.E., Hopkins J., Ingham T. (2007). Free radical modelling studies during the UK TORCH Campaign in Summer 2003. Atmos. Chem. Phys..

[70] Anderson D.C., Pavelec J., Daube C., Herndon S.C., Knighton W.B., Lerner B.M., Roscioli J.R., Yacovitch T.I., Wood E.C. (2019). Characterization of ozone production in San Antonio, Texas, using measurements of total peroxy radicals. Atmos. Chem. Phys..

[71] Tan Z.F., Lu K.D., Hofzumahaus A., Fuchs H., Bohn B., Holland F., Liu Y.H., Rohrer F., Shao M., Sun K. (2019). Experimental budgets of OH, HO_2_, and RO_2_ radicals and implications for ozone formation in the Pearl River Delta in China 2014. Atmos. Chem. Phys..

[72] Rohrer F., Berresheim H. (2006). Strong correlation between levels of tropospheric hydroxyl radicals and solar ultraviolet radiation. Nature.

[73] Ma W., Feng Z., Zhan J., Liu Y., Liu P., Liu C., Ma Q., Yang K., Wang Y., He H. (2022). Influence of photochemical loss of volatile organic compounds on understanding ozone formation mechanism. Atmos. Chem. Phys..

[74] Jenkin M.E., Valorso R., Aumont B., Rickard A.R. (2019). Estimation of rate coefficients and branching ratios for reactions of organic peroxy radicals for use in automated mechanism construction. Atmos. Chem. Phys..

[75] Cho C.M., Fuchs H., Hofzumahaus A., Holland F., Bloss W.J., Bohn B., Dorn H.P., Glowania M., Hohaus T., Liu L. (2023). Experimental chemical budgets of OH, HO_2_, and RO_2_ radicals in rural air in western Germany during the JULIAC campaign 2019. Atmos. Chem. Phys..

[76] Whalley L.K., Furneaux K.L., Goddard A., Lee J.D., Mahajan A., Oetjen H., Read K.A., Kaaden N., Carpenter L.J., Lewis A.C. (2010). The chemistry of OH and HO_2_ radicals in the boundary layer over the tropical Atlantic Ocean. Atmos. Chem. Phys..

[77] Zhang K., Huang L., Li Q., Huo J.T., Duan Y.S., Wang Y.H., Yaluk E., Wang Y.J., Fu Q.Y., Li L. (2021). Explicit modeling of isoprene chemical processing in polluted air masses in suburban areas of the Yangtze River Delta region: Radical cycling and formation of ozone and formaldehyde. Atmos. Chem. Phys..

[78] Liu T.T., Hong Y.W., Li M.R., Xu L.L., Chen J.S., Bian Y.H., Yang C., Dan Y.B., Zhang Y.N., Xue L.K. (2022). Atmospheric oxidation capacity and ozone pollution mechanism in a coastal city of southeastern China: Analysis of a typical photochemical episode by an observation-based model. Atmos. Chem. Phys..

[79] Civan M.Y., Elbir T., Seyfioglu R., Kuntasal Ö.O., Bayram A., Doğan G., Yurdakul S., Andiç Ö., Müezzinoğlu A., Sofuoglu S.C. (2015). Spatial and temporal variations in atmospheric VOCs, NO_2_, SO_2,_ and O_3_ concentrations at a heavily industrialized region in Western Turkey, and assessment of the carcinogenic risk levels of benzene. Atmos. Environ..

[80] Chan L.Y., Chu K.W., Zou S.C., Chan C.Y., Wang X.M., Barletta B., Blake D.R., Guo H., Tsai W.Y. (2006). Characteristics of nonmethane hydrocarbons (NMHCs) in industrial, industrial-urban, and industrial-suburban atmospheres of the Pearl River Delta (PRD) region of south China. J. Geophys. Res. Atmos..

[81] Duan J.C., Tan J.H., Yang L., Wu S., Hao J.M. (2008). Concentration, sources and ozone formation potential of volatile organic compounds (VOCs) during ozone episode in Beijing. Atmos. Res..

[82] Barletta B., Meinardi S., Rowland F.S., Chan C.Y., Wang X.M., Zou S.C., Chan L.Y., Blake D.R. (2005). Volatile organic compounds in 43 Chinese cities. Atmos. Environ..

[83] Kesselmeier J., Staudt M. (1999). Biogenic Volatile Organic Compounds (VOC): An Overview on Emission, Physiology and Ecology. J. Atmos. Chem..

[84] Guo H. (2011). Source apportionment of volatile organic compounds in Hong Kong homes. Build. Environ..

[85] Zheng J.Y., Yu Y.F., Mo Z.W., Zhang Z., Wang X.M., Yin S.S., Peng K., Yang Y., Feng X.Q., Cai H.H. (2013). Industrial sector-based volatile organic compound (VOC) source profiles measured in manufacturing facilities in the Pearl River Delta, China. Sci. Total Environ..

[86] McCarthy M.C., Aklilu Y.A., Brown S.G., Lyder D.A. (2013). Source apportionment of volatile organic compounds measured in Edmonton, Alberta. Atmos. Environ..

[87] Liu Y., Shao M., Fu L.L., Lu S.H., Zeng L.M., Tang D.G. (2008). Source profiles of volatile organic compounds (VOCs) measured in China: Part I. Atmos. Environ..

[88] Guo H., Wang T., Louie P.K.K. (2004). Source apportionment of ambient non-methane hydrocarbons in Hong Kong: Application of a principal component analysis/absolute principal component scores (PCA/APCS) receptor model. Environ. Pollut..

[89] Liu P.W.G., Yao Y.C., Tsai J.H., Hsu Y.C., Chang L.P., Chang K.H. (2008). Source impacts by volatile organic compounds in an industrial city of southern Taiwan. Sci. Total Environ..

[90] Juráň S., Karl T., Ofori-Amanfo K.K., Šigut L., Zavadilová I., Grace J., Urban O. (2025). Drought shifts ozone deposition pathways in spruce forest from stomatal to non-stomatal flux. Environ. Pollut..

[91] Zheng J.Y., Shao M., Che W.W., Zhang L.J., Zhong L.J., Zhang Y.H., Streets D.G. (2009). Speciated VOC emission inventory and spatial patterns of ozone formation potential in the Pearl River Delta, China. Environ. Sci. Technol..

[92] Liu Y., Shao M., Lu S., Chang C.C., Wang J.L., Chen G. (2008). Volatile Organic Compound (VOC) measurements in the Pearl River Delta (PRD) region, China. Atmos. Chem. Phys..

[93] Yu D., Tan Z.F., Lu K.D., Ma X.F., Li X., Chen S.Y., Zhu B., Lin L.L., Li Y.T., Qiu P.P. (2020). An explicit study of local ozone budget and NO*_x_*-VOCs sensitivity in Shenzhen China. Atmos. Environ..

[94] Tan Z.F., Lu K.D., Jiang M.Q., Su R., Wang H.L., Lou S.R., Fu Q.Y., Zhai C.Z., Tan Q.W., Yue D.L. (2019). Daytime atmospheric oxidation capacity in four Chinese megacities during the photochemically polluted season: A case study based on box model simulation. Atmos. Chem. Phys..

[95] Ainsworth E.A., Yendrek C.R., Sitch S., Collins W.J., Emberson L.D. (2012). The Effects of Tropospheric Ozone on Net Primary Productivity and Implications for Climate Change. Annu. Rev. Plant Biol..

[96] Crutzen P.J. (1974). Photochemical reactions initiated by and influencing ozone in unpolluted tropospheric air. Tellus A Dyn. Meteorol. Oceanogr..

[97] Li K.W., Wang X.H., Li L.M., Wang J., Liu Y.Y., Cheng X., Xu B., Wang X.Y., Yan P., Li S.J. (2021). Large variability of O_3_-precursor relationship during severe ozone polluted period in an industry-driven cluster city (Zibo) of North China Plain. J. Clean. Prod..

[98] Li L.M., Zheng Z.S., Xu B., Wang X.H., Bai Z.P., Yang W., Geng C.M., Li K.W. (2023). Investigation of O_3-_precursor relationship nearby oil fields of Shandong, China. Atmos. Environ..

[99] Steinfeld J.I. (1998). Atmospheric Chemistry and Physics: From Air Pollution to Climate Change. Environ. Sci. Policy Sustain. Dev..

[100] Tang X.Y., Zhang Y.H., Shao M. (2006). Atmospheric Environmental Chemistry.

[101] Zheng Z.S., Dou J.P., Zhang G.T., Li L.M., Xu B., Yang W., Bai Z.P. (2023). Photochemical Mechanism and Control Strategy Optimization for Summertime Ozone Pollution in an Industrial City in the North China Plain. Environ. Sci..

