# Supplementary material for: Unraveling the Drivers of Continuous Summer Ozone Pollution Episodes in Bozhou, China: Toward Targeted Control Strategies
Source: Toxics. 2025 Dec 29;14(1):37. doi: 10.3390/toxics14010037 (PMC12845908; doi:10.3390/toxics14010037)
Supplement: Supplementary file 1 [file toxics-14-00037-s001.zip › toxics-4047832-supplementary.pdf]

Unraveling the drivers of continuous summer ozone pollution episodes in Bozhou, China: Toward targeted control strategies

Ke Wu[1, 2, 3], Xuezhong Wang[1], Dandan Zhang[1, 4], Hong Li[1], Fang Bi[1], Zhenhai Wu[1, 5*], Fanxiu Li[2], Wanghui Chu[1,6], Cong An[1]

1. State Key Laboratory of Environmental Criteria and Risk Assessment, Chinese Research Academy of Environmental Sciences, Beijing 100012, China
2. College of Chemistry and Environmental Engineering, Yangtze University, Jingzhou 434023, China
3. Institute of Surface-Earth System Science, School of Earth System Science, Tianjin University, Tianjin 300072, China
4. College of Mechanical Engineering, Beijing Institute of Petrochemical Technology, Beijing 102617, China
5. Environmental Standard Institute, Ministry of Ecology and Environment, Chinese Research Academy of Environmental Sciences, Beijing 100012, China
6. Chinese Academy for Environmental Planning, Beijing 100012, China

*Corresponding authors.

E-mail: wuzh01@craes.org.cn (Zhenhai Wu)

Text S1 Error estimation analysis of PMF results

Setting and diagnosing the model operation using Q-values and e_{if}/σ_{if}, where Q-values should be close to the number of elements in the X matrix under reasonable error estimates. $Q_{(true)}$ should not exceed 1.5 times $Q_{(robust)}$, with $Q_{(true)}/Q_{(robust)}$ around 1.02. Otherwise, this indicates that outliers severely affect the assumption of normality in the data. For factor numbers of 4, 5, 6, and 7, $Q_{(true)}/Q_{(robust)}$ values are 1.05, 1.03, 1.01, and 1.01, respectively. The e_{if}/σ_{if} ratio represents the fit between the results and the original data. Residuals from PMF analysis are mostly within the range of -3.0 to 3.0, and as the number of factors is adjusted, the results stabilize (USEPA, 2014). After multiple runs of PMF, five factors were ultimately determined, with most compounds having an $R^2>$ 0.9 between observed and simulated values, indicating that five factors effectively explain the source information contained in the original data.

Text S2 Online monitoring of VOCs: sampling, analysis, and quality control

Ambient air was drawn into a sampling system, then passed through a preconcentration unit where analytes were cryogenically trapped on a capillary trap column. The trap was rapidly heated for thermal desorption, and the desorbed compounds entered the analytical system, were separated on the chromatographic column, and detected by GC–FID and GC–MS. The GC–FID channel quantified C_2–C_5 hydrocarbons, while the GC–MS channel identified and quantified C_5–C_{12} hydrocarbons, halogenated VOCs (HVOCs), oxygenated VOCs (OVOCs), and nitrogen-containing VOCs (NVOCs). The system operated automatically for 24 h with a time resolution of 60 min, including a 5 min sampling interval, yielding 23 samples per day. Detection limits for individual VOCs species ranged from 0.02×10^{-9} to 0.07×10^{-9}. To ensure data quality, a single-point calibration was performed daily at 00:00 LT using a 4×10^{-9} ppbv PAMS standard gas (Linde) to correct for retention-time drift. Multi-point calibrations and blank experiments were conducted at least monthly using TO-15 (Linde) and PAMS standards, achieving calibration R^2 values above 0.99. All sampling frequencies, durations, quality assurance, and quality control procedures complied with HJ/T 193-2005 ("Automated Methods for Ambient Air Quality Monitoring") and HJ 1010-2018 ("Continuous Monitoring System Specifications and Test Procedures for VOCs by GC"). Data capture efficiency exceeded 80%, with 115 valid species observed: 29 alkanes, 11 alkenes, 1 alkyne, 17 aromatics, 35 HVOCs, 21 OVOCs, and 1 sulfur-containing VOC. Notably, acetaldehyde concentrations remained consistently elevated during the monitoring period. This likely reflects contributions from both primary emissions, such as vehicle exhaust and industrial sources, and secondary photochemical formation via hydrocarbon oxidation. In contrast, ethanol is not included among the target VOCs in HJ/T 193-2005 and HJ 1010-2018. Although it can be partially detected by the system, its measured

concentrations were generally below the method detection limit (MDL). This limitation is important for interpreting the VOC dataset, as actual ethanol emissions may be underestimated.

Text S3 Model initialization and boundary layer assumptions in F0AM

To initialize the model, volume mixing ratios of key ozone precursors—such as VOCs, NO, NO_2, CO, and O_3—must be provided. Reaction rates and diffusion processes are influenced by meteorological parameters. F0AM does not account for horizontal or vertical transport. Instead, it simulates dilution within the mixed layer by applying a fixed dilution rate of 1.1/86400 s^{-1}, which reflects average conditions based on similar past studies (Li et al., 2021; Zheng et al., 2023; Li et al., 2023). It should be noted that using a fixed dilution rate is a simplification that may not fully capture the spatial and temporal variability of atmospheric transport, especially under stagnant conditions with low wind speeds, where ozone accumulation could be underestimated. For boundary conditions, background concentrations of key species are derived from rural monitoring sites outside Bozhou and introduced into the model at the same dilution rate to simulate the influence of background air. This approach is particularly important for long-lived species such as O_3, CO, and CH_4, while concentrations of more reactive, short-lived compounds are mainly influenced by local emissions and chemistry. It is important to acknowledge the limitations of the F0AM simulation. First, the model does not explicitly account for changes in boundary layer dynamics, which significantly affect vertical mixing and pollutant distribution, especially during transitions between day and night. Second, deposition is simplified as a uniform dilution process and not independently parameterized, which may introduce uncertainties in simulating the diurnal variation of pollutant concentrations and radical budgets.

Text S4 Mechanisms of O_3 Formation and Depletion in Ambient Air

1.O_3 Formation Mechanisms

O_3 formation and removal in the troposphere involve a network of complex chemical and physical processes. Near-surface O_3 is predominantly generated through photochemical reactions, serving as a key indicator of photochemical smog pollution (Wang et al., 2017) Its formation is largely controlled by two interlinked reaction cycles: the NO_x cycle and the RO_x cycle.

In the NO_x cycle, nitric oxide (NO) is oxidized to nitrogen dioxide (NO_2) catalyzed by peroxy radicals such as HO_2 and RO_2 (Eq. 1–2). NO_2 then undergoes photolysis under sunlight, releasing singlet oxygen $O(^3P)$ (Eq. 3), which reacts with O_2 to form O_3 (Eq. 4), representing the primary chemical pathway for tropospheric O_3 formation (Tang et al., 2006). Notably, O_3 is produced without net consumption of NO_x.

The RO_x cycle is initiated by the reaction of hydroxyl radicals (OH) with volatile organic compounds (VOCs), producing RO_2 radicals (Eq. 5). Through subsequent reactions (Eq. 2–4), O_3 is generated alongside RO radicals, which are ultimately converted to HO_2 (Eq. 6) and regenerated to OH in Eq. 1 (Wang et al., 2017). These processes illustrate the nonlinear interactions between O_3 and its precursors (Steinfeld, 1998; Xue et al., 2014; Wang et al., 2017).

$$HO_2 + NO \rightarrow OH + NO_2 \quad (1)$$

$$RO_2 + NO \rightarrow RO + NO_2 \quad (2)$$

$$NO_2 + hv \rightarrow NO + O(^3P) \quad (3)$$

$$O(^3P) + O_2 + M \rightarrow O_3 + M \quad (4)$$

$$OH + RH + O_2 \rightarrow RO_2 + H_2O \quad (5)$$

$$RO + O_2 \rightarrow HO_2 + \text{ carbonyls} \quad (6)$$

2. O_3 Depletion Mechanisms

O_3 removal in the atmosphere primarily occurs via gas-phase and heterogeneous reactions. In the gas phase, O_3 is consumed through reactions with radicals and other atmospheric constituents, particularly HO_2 and RO_2 (Eq. 7–11) (Crutzen, 1974). These reactions constitute the major chemical pathways for tropospheric O_3 depletion.

Heterogeneous processes also contribute significantly. In the aqueous phase, O_3 reacts with sulfur dioxide (SO_2) and NO_2 to form sulfate and nitrate, accounting for approximately one-third of total O_3 removal (Tang et al., 2006). Additionally, O_3 can be deposited into plant tissues via stomatal uptake, representing another important atmospheric sink (Ainsworth et al., 2012).

$$O_3 + H_2O(g) + hv \rightarrow O_2 + 2OH \quad (7)$$

$$O_3 + OH \rightarrow HO_2 + O_2 \quad (8)$$

$$O_3 + HO_2 \rightarrow OH + 2O_2 \quad (9)$$

$$O_3 + NO \rightarrow NO_2 + O_2 \quad (10)$$

$$O_3 + NO_2 \rightarrow NO_3 + O_2 \quad (11)$$

Text S5 Applicability and limitations of the OFP method and complementarity with RIR analysis

The ozone formation potential (OFP) method adopted in this study is based on the maximum incremental reactivity (MIR) scale developed by Carter (1994) in which MIR values were derived from emission-based zero-dimensional modeling scenarios under high-NO_x conditions where ozone formation is maximally sensitive to VOCs. When applied to ambient VOC concentrations, several methodological considerations arise. First, because MIR coefficients are defined relative to emissions, OFP derived from ambient concentrations may underestimate the contribution of short-lived highly reactive compounds due to their rapid atmospheric removal prior to accumulation in the ambient air. Second, MIR represents ozone sensitivity under high- NO_x conditions, whereas Bozhou frequently experiences transitional or NO_x-limited regimes, meaning that MOIR or EBIR reactivity scales could theoretically be more representative of such environments.

Despite these limitations, OFP remains a widely used diagnostic metric for evaluating the relative contribution of VOC species to potential ozone formation and provides an intuitive means of comparing VOC groups. In the present study, OFP results are therefore interpreted as a relative indicator rather than an absolute quantitative predictor of ozone production.

To better reflect site-specific atmospheric chemistry, we further applied a zero-dimensional observation-based model (OBM) to calculate relative incremental reactivity (RIR), which quantifies the sensitivity of ozone production to perturbations in precursor concentrations under locally constrained meteorological and chemical conditions. RIR therefore complements the OFP framework by representing process-based chemical sensitivity, while OFP highlights species-level contribution tendencies. The combined application of OFP and RIR provides a more robust and comprehensive assessment of VOC–O_3 formation relationships in Bozhou.

Table S1 Presents the hourly measurements of trace gas concentrations, $PM_{2.5}$ levels, meteorological parameters, and total volatile organic compounds (TVOCs) recorded at the Bozhou University site during the period from June 8 th to June 16 th, 2023.

Species/parameter	Mean±SD	Median	Max
O_3(ppbv)	77.1±26.1	78.7	152.7
NO_2(ppbv)	5.56±2.8	5.0	19.4
NO(ppbv)	0.4±0.5	0.0	2.5
SO_2(ppbv)	2.7±0.9	2.4	8.6
CO(ppmv)	0.5±0.1	0.5	1.1
TVOCs(ppbv)	19.8±6.9	18.1	49.2
$PM_{2.5}$(μg/m^3)	23.5±11.5	21.0	67.0
RH (%)	47.7±17.3	46.5	88.0
TEMP (℃)	29.8±4.1	30.0	38.6

Table S2 VOCs species and the corresponding method detection limits (MDL, unit: ppbv) and MIR values.

VOCs species	MDL	MIR	VOCs species	MDL	MIR
**Alkanes (PAMS species)**			cis-2-Butene	0.046	14.24
Ethane	0.081	0.28	1-Pentene	0.022	7.21
Propane	0.031	0.49	trans-2-Pentene	0.034	10.56
n-Hexane	0.022	1.24	Isoprene	0.028	10.61
2,3-Dimethylpentane	0.041	1.34	cis-2-Pentene	0.034	10.38
Cyclohexane	0.022	1.25	1-Hexene	0.031	5.49
n-Heptane	0.028	1.07	**Alkyne (PAMS species)**		
n-Nonane	0.046	0.78	Acetylene	0.058	0.95
n-Undecane	0.092	0.61	**Aromatics (PAMS species)**		
n-Butane	0.023	1.15	Benzene	0.037	0.72
i-Pentane	0.044	1.45	Styrene	0.060	1.73
n-Pentane	0.029	1.31	Isopropylbenzene	0.052	2.52
2,2-Dimethylbutane	0.025	1.17	*m*-Ethyltoluene	0.064	8.87
Cyclopentane	0.085	2.39	*p*-Ethyltoluene	0.099	11.76
2,3-Dimethylbutane	2.986	0.97	1,3,5-Trimethylbenzene	0.084	4.44
2-Methylpentane	0.061	1.50	Toluene	0.056	4.00
3-Methylpentane	0.067	1.80	Ethylbenzene	0.050	3.04
2,4-Dimethylpentane	0.013	1.55	*m/p*-Xylene	0.049	7.80
Methylcyclopentane	0.093	2.19	*o*-Xylene	0.055	7.64
2-Methylhexane	0.021	1.19	*n*-Propylbenzene	0.076	2.03
3-Methylhexane	0.025	1.61	1,2,3-Trimethylbenzene	0.081	11.97
2,2,4-Trimethylpentane	0.030	1.26	*m*-Diethylbenzene	0.092	7.10
Methylcyclohexane	0.031	1.70	*p*-Diethylbenzene	0.086	4.43
2,3,4-Trimethylpentane	0.053	1.22	*o*-Ethyltoluene	0.080	5.59
2-Methylheptane	0.050	1.07	1,2,4-Trimethylbenzene	0.075	8.87
3-Methylheptane	0.052	1.24	**OVOCs**		
n-Octane	0.046	0.90	Acetaldehyde	0.053	6.54
n-Decane	0.083	0.68	Acrolein	0.121	7.45
n-Dodecane	0.226	0.55	Propionaldehyde	0.082	7.08
i-Butane	0.044	1.23	Methacrolein	0.034	6.01
**Alkenes (PAMS species)**			*n*-Butyraldehyde	0.049	5.97
Ethene	0.069	9.00	Crotonaldehyde	0.049	9.39
Propylene	0.098	11.66	Valeraldehyde	0.073	5.08
1,3-Butadiene	0.034	12.61	Hexanal	0.126	4.35
1-Butene	0.032	9.73	Acetone	0.040	0.36
trans-2-Butene	0.049	15.16			

Table S3 VOC concentration measurements in Bozhou from June 8th to 16th, 2023 (units: pptv).

VOCs species	Mean ± SD	VOCs species	Mean ± SD	VOCs species	Mean ± SD
**Alkanes**		trans-2-Pentene	3±3	1,1-Dichloroethane	13±7
Ethane	2036±590	1-Pentene	13±7	Chloroform	210±250
Propane	1336±714	cis-2-Pentene	3±3	1,1,1-Trichloroethane	4±2
Isobutane	319±132	Isoprene	339±483	1,2-Dichloroethane	284±130
n-Butane	440±234	**Alkyne**		Trichloroethylene	6±6
Isopentane	2072±3635	Acetylene	758±316	1,2-Dichloropropane	86±32
n-Pentane	160±85	**Aromatics**		1,1,2-Trichloroethane	10±3
2,2-Dimethylbutane	34±19	Benzene	726±166	Tetrachloroethylene	12±14
2,3-Dimethylbutane	44±20	Toluene	129±77	1,2-Dibromoethane	2±2
2-Methylpentane	119±58	Ethylbenzene	36±37	**OVOCs**	
3-Methylpentane	75±33	Styrene	13±17	Acetaldehyde	3091±1096
n-Hexane	29±16	*o*-Xylene	20±23	*n*-Butyraldehyde	187±177
Cyclohexane	33±25	Isopropylbenzene	3±5	Acrolein	296±290
2-Methylhexane	12±10	Propylbenzene	3±4	Propionaldehyde	463±209
3-Methylhexane	13±11	1-Ethyl-3-methylbenzene	3±4	Acetone	1833±730
n-Heptane	10±9	*p*-Ethyltoluene	3±9	Methyl tert-butyl ether	62±27
n-Octane	14±26	1,3,5-Trimethylbenzene	1±1	Methacrolein	130±108
n-Nonane	6±12	1-Ethyl-2-methylbenzene	2±3	Valeraldehyde	27±28
n-Undecane	1±1	1,2,4-Trimethylbenzene	2±1	Caproaldehyde	20±12
n-Dodecane	1±1	1,2,3-Trimethylbenzene	2±7	Isopropanol	17±12
**Alkenes**		*p*-Diethylbenzene	2±1	Vinyl acetate	29±16
1-Hexene	7±5	Halocarbons		Ethyl acetate	27±26
Ethene	1033±1324	Methyl chloride	814±435	Methyl ethyl ketone	56±31
Propene	137±141	Vinyl chloride	6±12	Methyl isobutyl ketone	4±3
trans-2-Butene	6±9	Ethyl chloride	32±28	2-Hexanone	9±3
1-Butene	91±49	1,1-Dichloroethylene	5±12	Benzaldehyde	102±34
cis-2-Butene	9±7	Methylene chloride	643±235	*m*-Tolualdehyde	22±15

Table S4 Summary of model reaction fluxes: Average reaction rates (ppbv/h) of radical formation, cross-reactions, and termination throughout the entire reaction process.

Reaction rate (ppbv/h)		Beijing suburban (Ma et al., 2022)	Changzhou suburban (Zhang et al., 2021)	Dongying suburban (Chen et al., 2020)	HongKong suburban (Xue et al., 2016)	Xiamen urban (Liu et al., 2022)	Huaibei suburban (Wei et al., 2023)	This work
Primary radical production	O_3+hv→OH	0.3	0.5	1.2	0.9	1.4	0.4	0.8
	HONO+hv→OH	1.3	0.3	0.5	1.5	1.3	0.3	0.2
	Alkenes+O_3→OH	0.1	-	0.3	0.2	0	0.1	0
	HCHO+hv→HO_2	0.3	0.5	1.1	1.2	0.9	0.6	0.6
	OVOCs+hv→HO_2	0.4	0.7	1	1.3	0.4	0.4	0.3
	Alkenes+O_3→HO_2	0	-	0.2	0.1	0	0	0
	OVOCs+hv→RO_2	0.2	0.6	0.9	1.3	0.4	0.3	0.3
	Alkene+O_3→RO_2	0.1	-	0.1	0.1	0	0.1	0.1
Terminate processes	OH+NO	0.1	0.1	0.1	0.3	0.1	0.1	0
	OH+NO_2	1.3	2.4	0.5	3.5	2.4	0.4	0.6
	HO_2+HO_2	0.2	0.5	0.6	0.2	-	0.4	0.5
	HO_2+RO_2	0.3	0	1.1	0.1	0.1	0.7	0.7
	RO_2+RO_2	0.1	-	-	-	-	0	0
	RO_2+NO	0.1	0.4	0.1	0.4	0.2	0.1	0.1
	RO_2+NO_2	0.2	0.6	0.5	1.5	0.4	0.1	0.3
Radical propagation	OH+VOCs→RO_2	4	1.2	6.5	7.3	3	2.9	1.8
	OH+HCHO/CO/O_3→HO_2	0.5	3.5	2.6	4.3	4	1.7	2.6
	HO_2+NO→OH	4.9	8.3	7.6	13.4	7.9	5.2	6.9
	RO_2+NO→HO_2	3.2	3.3	5.1	6.7	2.4	3	2.4

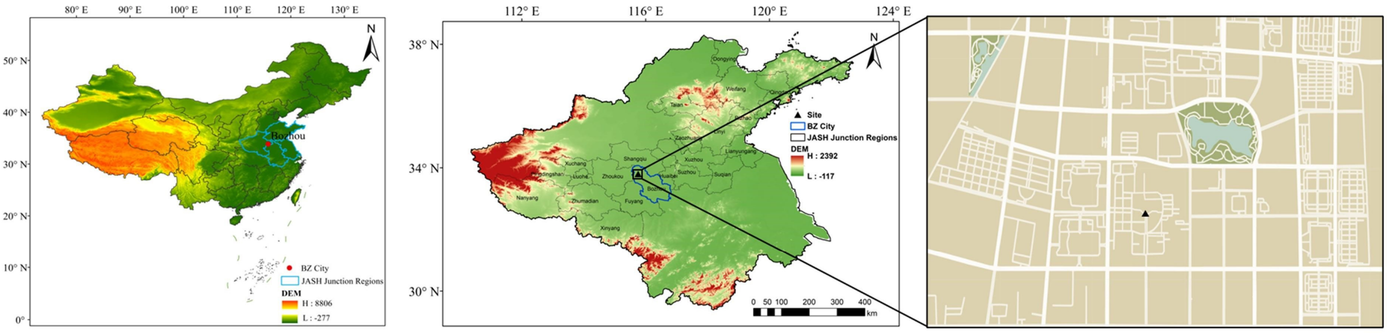

Figure S1 Map of monitoring sites in Bozhou City.

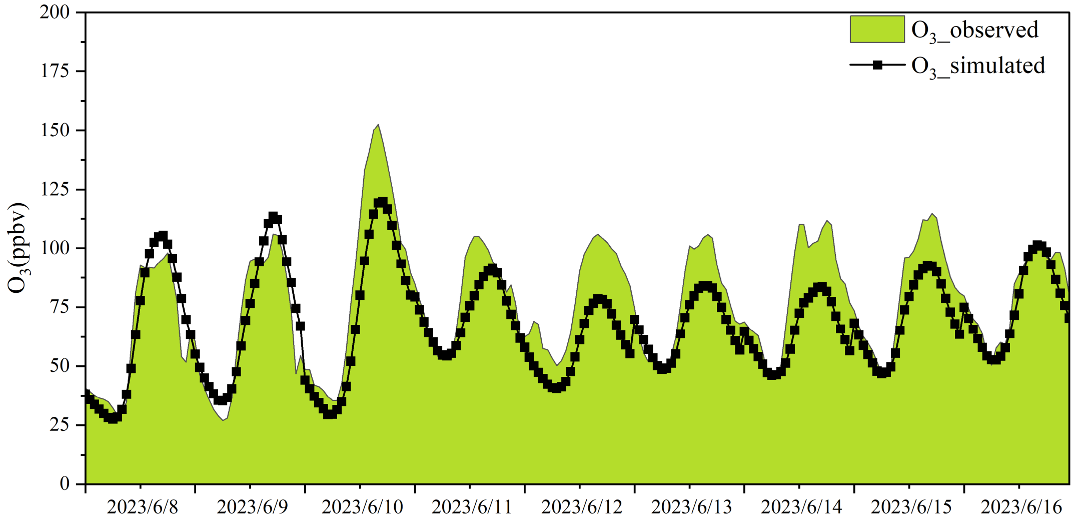

Figure S2 Comparison of simulated and observed O_3 at the Bozhou University site from June 8th to 16th, 2023.

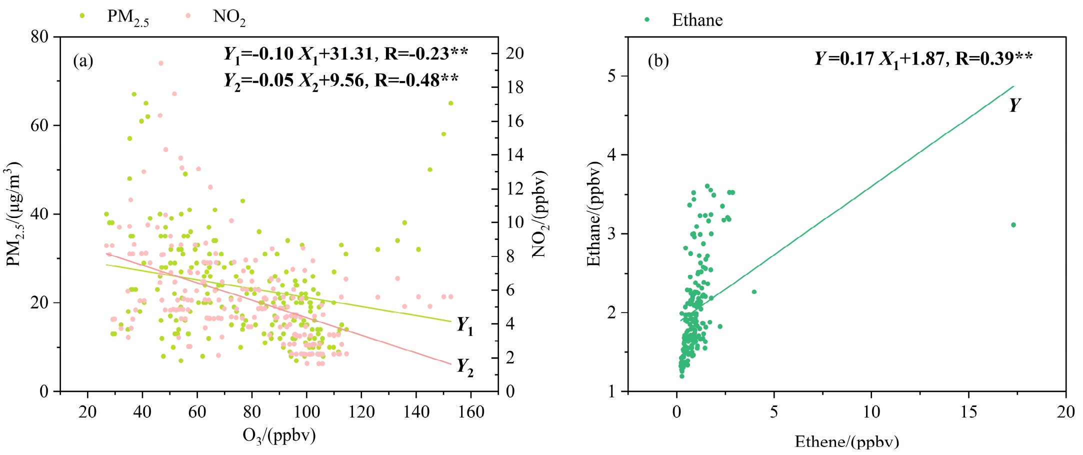

Figure S3 (a) Correlation of NO_2, $PM_{2.5}$, and O_3 concentrations at the Bozhou University site

(b) Correlation between ethylene and ethane.

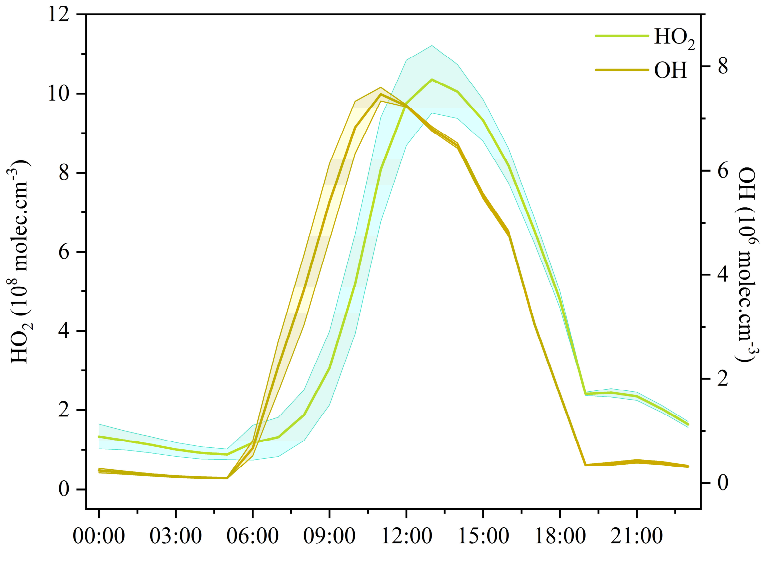

Figure S4 Average diurnal variation of OH and HO_2 during simulated O_3 pollution period at the Bozhou University site (shading represents the standard deviation of the mean across 6 simulations).

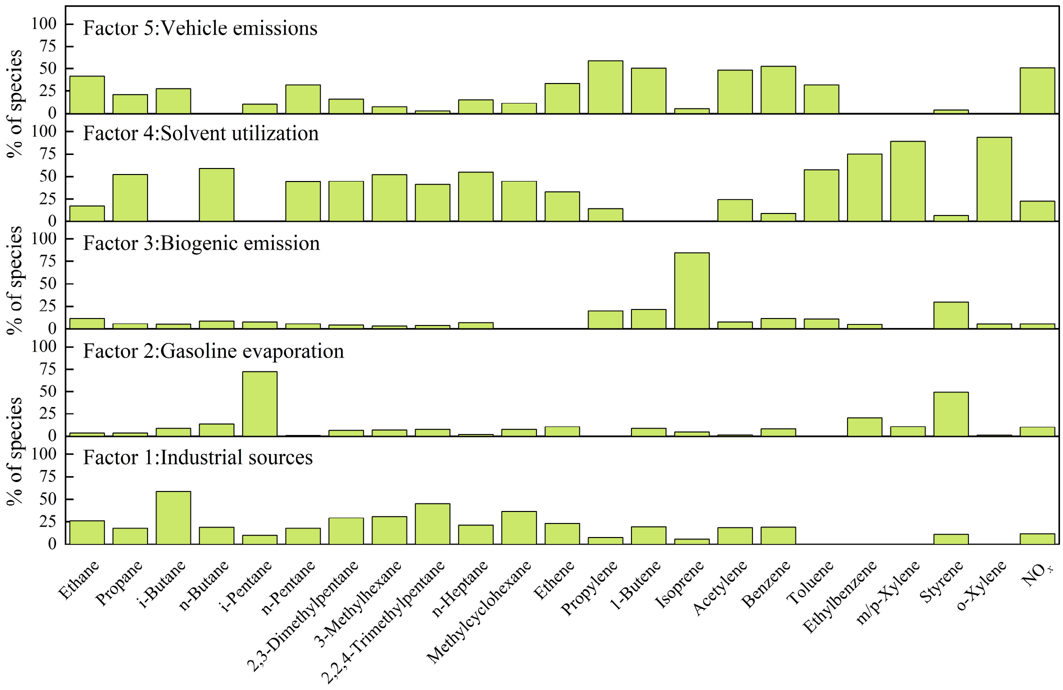

Figure S5 Contributions of various species to the Bozhou University site during the O_3 pollution period as analyzed by the PMF model.

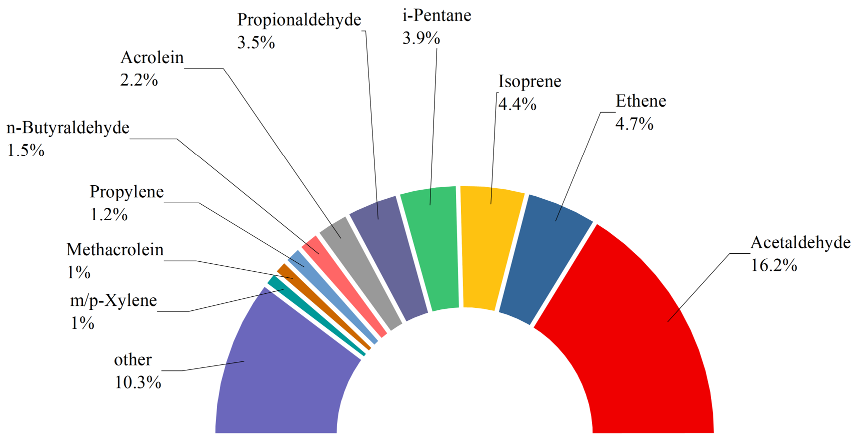

Figure S6 Top 10 species of OFP and their proportions at the Bozhou University site during the O_3 pollution period.

References

1. Ainsworth, E.A., et al., 2012. The Effects of Tropospheric Ozone on Net Primary Productivity and Implications for Climate Change. Annu. Rev. Plant Biol. 63, 637–661. https://doi.org/10.1146/annurev-arplant-042110-103829
2. Carter, W.P.L., 1994. Development of Ozone Reactivity Scales for Volatile Organic Compounds. Air & Waste 44, 881–899. https://doi.org/10.1080/1073161X.1994.10467290
3. Chen, T., et al., 2020. Volatile organic compounds and ozone air pollution in an oil production region in northern China. Atmos. Chem. Phys. 20, 7069–7086. https://doi.org/10.5194/acp-20-7069-2020.
4. Crutzen, P.J., 1974. Photochemical reactions initiated by and influencing ozone in unpolluted tropospheric air. Tellus A: Dynamic Meteorology and Oceanography 26, 47. https://doi.org/10.3402/tellusa.v26i1-2.9736
5. Li, K., et al., 2021. Large variability of O_3-precursor relationship during severe ozone polluted period in an industry-driven cluster city (Zibo) of North China Plain. J. Clean. Prod. 316, 128252. https://doi.org/10.1016/j.jclepro.2021.128252.
6. Li, L., et al., 2023. Investigation of O_3-precursor relationship nearby oil fields of Shandong, China. Atmos. Environ. 294, 119471. https://doi.org/10.1016/j.atmosenv.2022.119471.
7. Liu, T., et al., 2022. Atmospheric oxidation capacity and ozone pollution mechanism in a coastal city of southeastern China: analysis of a typical photochemical episode by an observation-based model. Atmos. Chem. Phys. 22, 2173–2190. https://doi.org/10.5194/acp-22-2173-2022.
8. Ma, W., et al., 2022. Influence of photochemical loss of volatile organic compounds on understanding ozone formation mechanism. Atmos. Chem. Phys. 22, 4841–4851. https://doi.org/10.5194/acp-22-4841-2022.
9. Steinfeld, J.I., 1998. Atmospheric Chemistry and Physics: From Air Pollution to Climate Change. Environment: Science and Policy for Sustainable Development 40, 26–26. https://doi.org/10.1080/00139157.1999.10544295
10. Tang, X., et al., 2006. Atmospheric Environmental Chemistry. Higher Education Press, Beijing.
11. USEPA, 2014. Positive Matrix Factorization (PMF) 5.0 Fundamentals and User Guide. USEPA Office of Research and Development.
12. Wang, T., et al., 2017. Ozone pollution in China: A review of concentrations, meteorological influences, chemical precursors, and effects. Sci. Total Environ. 575, 1582–1596. https://doi.org/10.1016/j.scitotenv.2016.10.081
13. Wei, N., et al., 2023. Peroxy radical chemistry during ozone photochemical pollution season at a suburban site in the boundary of Jiangsu–Anhui–Shandong–Henan region, China. Sci. Total Environ. 904, 166355. https://doi.org/10.1016/j.scitotenv.2023.166355.
14. Xue, L.K., et al.,2014. Increasing External Effects Negate Local Efforts to Control Ozone Air Pollution: A Case Study of Hong Kong and Implications for Other Chinese Cities. Environ. Sci. Technol. 48, 10769–10775. https://doi.org/10.1021/es503278g
15. Xue, L.K., et al., 2016. Oxidative capacity and radical chemistry in the polluted atmosphere of Hong Kong and Pearl River Delta region: analysis of a severe photochemical smog episode. Atmos. Chem. Phys. 16, 9891–9903. https://doi.org/10.5194/acp-16-9891-2016.
16. Zhang, K., et al., 2021. Explicit modeling of isoprene chemical processing in polluted air masses in suburban areas of the Yangtze River Delta region: radical cycling and formation of ozone and formaldehyde. Atmos. Chem. Phys. 21, 5905–5917. https://doi.org/10.5194/acp-21-5905-2021.
17. Zheng, Z.S., et al., 2023. Photochemical Mechanism and Control Strategy Optimization for Summertime Ozone Pollution in an Industrial City in the North China Plain. Environ. Sci. 44, 1821–1829. https://doi.org/10.13227/j.hjkx.202210204.
